# Diversity and Evolutionary History of Ti Plasmids of “tumorigenes” Clade of *Rhizobium* spp. and Their Differentiation from Other Ti and Ri Plasmids

**DOI:** 10.1093/gbe/evad133

**Published:** 2023-07-18

**Authors:** Nemanja Kuzmanović, Jacqueline Wolf, Sabine Eva Will, Kornelia Smalla, George C diCenzo, Meina Neumann-Schaal

**Affiliations:** Julius Kühn Institute (JKI), Federal Research Centre for Cultivated Plants, Institute for Plant Protection in Horticulture and Urban Green, Braunschweig, Germany; Leibniz Institute DSMZ-German Collection of Microorganisms and Cell Cultures, Braunschweig, Germany; Leibniz Institute DSMZ-German Collection of Microorganisms and Cell Cultures, Braunschweig, Germany; Julius Kühn Institute (JKI), Federal Research Centre for Cultivated Plants, Institute for Epidemiology and Pathogen Diagnostics, Braunschweig, Germany; Department of Biology, Queen's University, Kingston, Ontario, Canada; Leibniz Institute DSMZ-German Collection of Microorganisms and Cell Cultures, Braunschweig, Germany

**Keywords:** *Rhizobium tumorigenes*, *Rhizobium rhododendri*, classification, opine, ridéopine, leucinopine

## Abstract

Agrobacteria are important plant pathogens responsible for crown/cane gall and hairy root diseases. Crown/cane gall disease is associated with strains carrying tumor-inducing (Ti) plasmids, while hairy root disease is caused by strains harboring root-inducing (Ri) plasmids. In this study, we analyzed the sequences of Ti plasmids of the novel “tumorigenes” clade of the family *Rhizobiaceae* (“tumorigenes” Ti plasmids), which includes two species, *Rhizobium tumorigenes* and *Rhizobium rhododendri*. The sequences of reference Ti/Ri plasmids were also included, which was followed by a comparative analysis of their backbone and accessory regions. The “tumorigenes” Ti plasmids have novel opine signatures compared with other Ti/Ri plasmids characterized so far. The first group exemplified by pTi1078 is associated with production of agrocinopine, nopaline, and ridéopine in plant tumors, while the second group comprising pTi6.2 is responsible for synthesis of leucinopine. Bioinformatic and chemical analyses, including opine utilization assays, indicated that leucinopine associated with pTi6.2 most likely has D,L stereochemistry, unlike the L,L-leucinopine produced in tumors induced by reference strains Chry5 and Bo542. Most of the “tumorigenes” Ti plasmids have conjugative transfer system genes that are unusual for Ti plasmids, composed of *avhD4*/*avhB* and *traA*/*mobC*/*parA* regions. Next, our results suggested that “tumorigenes” Ti plasmids have a common origin, but they diverged through large-scale recombination events, through recombination with single or multiple distinct Ti/Ri plasmids. Lastly, we showed that Ti/Ri plasmids could be differentiated based on pairwise Mash or average amino-acid identity distance clustering, and we supply a script to facilitate application of the former approach by other researchers.

**Significance**

Tumor-inducing (Ti) plasmids are replicons indispensable for the pathogenicity of agrobacteria, and it is important to better understand their diversity and evolution. Here, we analyzed Ti plasmids of the “tumorigenes” clade of the family *Rhizobiaceae*, which differ from other Ti plasmids characterized so far. We infer from our analyses that “tumorigenes” Ti plasmids have a common origin, but they diverged through large-scale recombination events, through recombination with single or multiple distinct Ti and root-inducing (Ri) plasmids. We have also prepared a pipeline that facilitates grouping of Ti and related Ri plasmids. Our results shed new light on the evolution, diversification, and classification of Ti plasmids, which might impact future studies on epidemiology, ecology, and diagnostics of crown gall.

## Introduction

Agrobacteria are widespread plant pathogens affecting various agricultural crops. They are responsible for economically important diseases, including crown gall, cane gall, and hairy root (also known as root mat or crazy root) (Escobar and Dandekar 2003; Otten et al. 2008; Bosmans et al. 2017). Agrobacteria are a taxonomically diverse group of bacteria, with representatives in several genera of the family *Rhizobiaceae*, primarily in the genera *Agrobacterium*, *Allorhizobium*, and *Rhizobium* (de Lajudie et al. 2019).

Agrobacteria contain multipartite genomes, consisting of a main chromosome, chromid(s), and (mega)plasmid(s) (Harrison et al. 2010; Slater et al. 2013; Kuzmanović et al. 2023). Particularly important are oncogenic plasmids that are indispensable for the pathogenicity of agrobacteria. They are divided into two classes: tumor-inducing (Ti) and root-inducing (Ri) plasmids (Suzuki et al. 2009). The former class is associated with crown gall and cane gall diseases, while the latter is harbored by strains causing hairy root disease.

Ti and Ri plasmids show a mosaic structure, whose evolution was driven by extensive horizontal gene transfer (HGT) and recombination events (Otten et al. 1992; Weisberg et al. 2020). Consequently, Ti and Ri plasmids are highly variable in size and structure. To be classified as Ti or Ri plasmid, a replicon must possess so-called transferred DNA (T-DNA), virulence (*vir*) genes, and opine catabolism genes. Infection of plants by agrobacteria involves transfer of T-DNA into the host cell nucleus and its integration into the host genome, through a process mediated by the *vir* genes and their products (Gelvin 2017). T-DNA genes are expressed in plants and can be divided into oncogenes and opine-related genes. Oncogenes can be further subdivided into genes associated with synthesis of plant hormones (auxin and cytokinin) and so-called *plast* genes (for phenotypic plasticity) (Britton et al. 2008; Otten 2018). Opine-related genes are associated with the production of specific organic compounds called opines, which are primarily used as selective nutrient sources by the pathogen, although some can also act as inducers of the conjugative transfer of the Ti plasmid (Dessaux et al. 1998; Farrand 1998; Dessaux and Faure 2018a).

Additionally, Ti/Ri plasmids carry backbone regions involved in replication and partitioning and conjugative transfer. As is true for most of the large plasmids present in the family *Rhizobiaceae*, Ti/Ri plasmids belong to the *repABC* plasmid family (Farrand 1998; Pinto et al. 2012). The conjugative transfer genes of the Ti/Ri plasmids characterized to date are organized into *tra* and *trb* operons, involved in the formation of the DNA transfer and replication (Dtr; or MOB) and mating pair formation (Mpf) complexes, respectively (Farrand 1998; Pappas and Cevallos 2011; Wetzel et al. 2015). Conjugal transfer systems are especially well studied in Ti plasmids. As mentioned above, their conjugal transfer is induced by opines and regulated by a quorum-sensing (QS) system (Farrand 1998; White and Winans 2007; Faure and Lang 2014; Dessaux and Faure 2018b).

The mosaic structure of Ti/Ri plasmids makes their classification and grouping difficult. Traditionally, Ti/Ri plasmids, including their host strains, were classified according to the type of opine(s) produced in the tumors they induce (Petit et al. 1983; Szegedi et al. 1988; Dessaux et al. 1998; Otten et al. 2008). Although opines play a critical role in the ecology of agrobacteria (Dessaux and Faure 2018a), genes involved in opine synthesis encompass a relatively small portion of Ti/Ri plasmids (Otten et al. 2008), and classification based on this trait does not consider other plasmid regions.

During the first decade of 21th century, only a few complete Ti/Ri plasmids were sequenced, including pTiOctopine (Zhu et al. 2000), pTi-SAKURA (Suzuki et al. 2000), pTiBo542 (Oger et al. 2001), pRi1724 (Moriguchi et al. 2001), and pTiC58 and pTiS4 (Slater et al. 2009). The expansion of next-generation sequencing (NGS) technologies led to an increase in the availability of whole-genome sequences of agrobacteria, which has allowed for more thorough comparative genomic analyses of their Ti/Ri plasmids. In this respect, Otten (2021) analyzed T-DNA regions from 350 agrobacterial genomes and classified them into three main groups, which were further divided into a number of subgroups. Nabi et al. (2022) investigated the diversity among T-DNAs and *vir* regions of 56 Ti/Ri plasmid sequences. In recent studies, complete sequences of several Ti/Ri plasmids and their relationship with related plasmids were investigated (Hooykaas and Hooykaas 2021a, 2021b; Shao et al. 2018; Kuzmanović and Puławska 2019; Shao et al. 2019). Furthermore, Weisberg et al. (2020) analyzed complete sequences of 143 Ti/Ri plasmids and classified them into nine distinct lineages (types) based on their evolutionary relationships, including six Ti (I–VI) and three Ri (I–III) types. Later, this list was expanded by the addition of five new Ti (VII–XI) types (Weisberg et al. 2022).

Although Ti/Ri plasmid sequences have been investigated in a number of studies, our knowledge on the natural host range, genetic diversity, and structure of these replicons should be further expanded. These data will contribute to a better understanding of evolution and ecology of these replicons. In our recent work, we characterized the novel “tumorigenes” clade of the family *Rhizobiaceae*, which includes tumorigenic bacteria *Rhizobium tumorigenes* and *Rhizobium rhododendri* isolated from crown gall tumors of blackberry and rhododendron, respectively (Kuzmanović et al. 2018; Kuzmanović et al. 2019; Kuzmanović et al. 2023). Analysis of draft genome sequences of the rhododendron strains suggested that they carry atypical Ti plasmids (Kuzmanović et al. 2019). We subsequently generated complete genome sequences of representative strains of *R. tumorigenes* (932 and 1078^T^) and *R. rhododendri* (rho-6.2^T^) (Kuzmanović et al. 2023). Here, we analyzed the sequences of plasmids pTi932, pTi1078, and pTi6.2, as well as related Ti plasmids of *R. rhododendri* strains originating from the United States. We collectively refer to these Ti plasmids as the “tumorigenes” Ti plasmids. Moreover, complete sequences of “tumorigenes” Ti plasmids were compared with previously described reference Ti/Ri plasmids, including comparative analysis of their backbone and accessory regions. The presence of diverse opine(s) in tumors induced by representative “tumorigenes” strains was verified by tandem mass spectrometry analysis. Lastly, we demonstrate an easy and convenient method for differentiation of Ti/Ri plasmids based on Mash- or average amino-acid identity (AAI)-distance clustering.

## Results

### Ti Plasmid Sequence Features and Functional Modules

The Ti plasmids pTi932, pTi1078, and pTi6.2 range in size from approximately 381 to 439 kb and are larger than typical Ti plasmids whose size are generally around 200 kb (supplementary table S1, Supplementary Material online). Related Ti plasmids carried by “tumorigenes” strains isolated in the United States are similar in size to each other, except for pTiL51/94 (∼280 kb) and pTiB230/85 (∼197 kb) (tables 1 and supplementary S1, Supplementary Material online). The guanine–cytosine (GC) content of the Ti plasmids carried by “tumorigenes” strains ranges from 55.8% to 56.3%, which is similar to the reference Ti plasmids (54.5–57.7%; tables 1 and supplementary S1, Supplementary Material online).

**Table 1:** General Features of “Tumorigenes” Ti Plasmids Investigated in This Study

Ti plasmid	Host bacterium	Plasmid Type^a^	Associated Opines^b^	Size (bp)	GC Content (%)	Accession Number	Reference
pTi932	*Rhizobium tumorigenes* 932	VII	Agrocinopine, nopaline, ridéopine	430,508	56.0	CP117263.1	Kuzmanović et al. (2023)
pTi1078	*Rhizobium tumorigenes* 1078^T^	VII	Agrocinopine, nopaline, ridéopine	439,071	56.2	CP117257.1	Kuzmanović et al. (2023)
pTiB21/90	*Rhizobium rhododendri* B21/90	VII	Agrocinopine, nopaline, ridéopine	455,093	56.3	CP072164.1	Weisberg et al. (2022)
pTiK1/93	*Rhizobium rhododendri* K1/93	VII	Agrocinopine, nopaline, ridéopine	420,487	56.2	CP072148.1	Weisberg et al. (2022)
pTiK15/93	*Rhizobium rhododendri* K15/93	VII	Agrocinopine, nopaline, ridéopine	420,652	56.2	CP072153.1	Weisberg et al. (2022)
pTi6.2	*Rhizobium rhododendri* rho-6.2^T^	VIIIa	Leucinopine	381,845	56.1	CP117269.1	Kuzmanović et al. (2023)
pTiL51/94	*Rhizobium rhododendri* L51/94	VIIIa	Leucinopine	279,225	55.8	CP072145.1	Weisberg et al. (2022)
pTiB230/85	*Rhizobium rhododendri* B230/85	VIIIb	Leucinopine	196,592	55.9	CP072160.1	Weisberg et al. (2022)

a Evolutionary classification proposed by Weisberg et al. (2020, 2022).

b Opines detected in tumors induced by strain carrying respective plasmid. Presence of opines in the tumors was experimentally demonstrated for plasmids pTi932, pTi1078, and pTi6.2 (this study). For other plasmids, prediction of opine production was based on bioinformatics analysis.

**Fig. 1.**
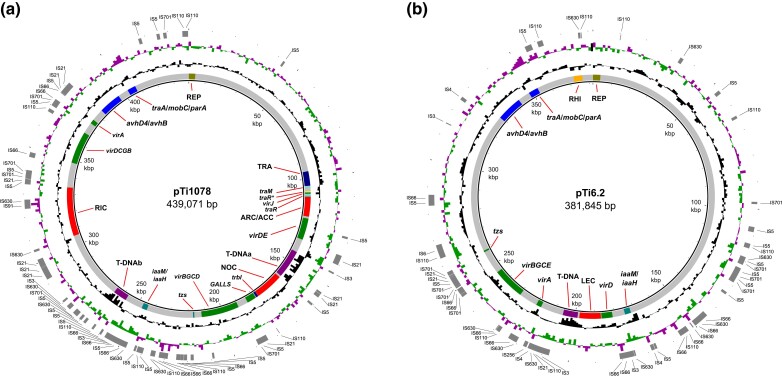
Circular maps of pTi1078 (*a*) and pTi6.2 (*b*). Each replicon is presented by a circular plot containing five rings. Genetic coordinates are shown within the thin inner ring. The next ring shows plasmid functional modules and specific genes as follows: replication-partitioning system (REP), conjugation systems (TRA, *avhD4*/*avhB* and *traA*/*mobC*/*parA*), virulence genes (*vir* and *GALLS*), T-DNA, agrocinopine regulation of conjugation of which regulatory gene *traR* is a member (ARC), which is linked to the transport and catabolism of agrocinopine region (ACC), transport and catabolism of nopaline (NOC), ridéopine (RIC), and leucinopine (LEC), non-T-DNA genes for biosynthesis of auxin (*iaaM/iaaH*) and cytokinin (*tzs*), *rhi* genes involved in QS (RHI). Genes *traM* and additional copy of *traR gene* (truncated) involved in regulation of conjugative transfer and gene which encodes conjugal transfer protein TrbI (*trbI*) are also shown. The next two rings portray GC content (black ring) and GC skew (purple/green). The outermost ring highlights IS elements identified using ISEscan (shown in gray). As plasmids pTi932 and pTi1078 have the identical organization of functional modules and exhibited high genetic relatedness, we show only the genetic map of pTi1078. The figure was generated using BRIG software and edited with Inkscape.

We identified different functional modules that could be divided into the backbone and accessory groups. Backbone regions include putative replication and partitioning and conjugation systems (fig. 1). Accessory regions include putative functional modules involved in pathogenesis, opine catabolism regions, and additional accessory regions or genes (fig. 1). More detailed analyses of each of these regions and genes are presented below.

### Differentiation of Ti/Ri Plasmids and Whole-Plasmid Sequence Comparisons

**Fig. 2.**
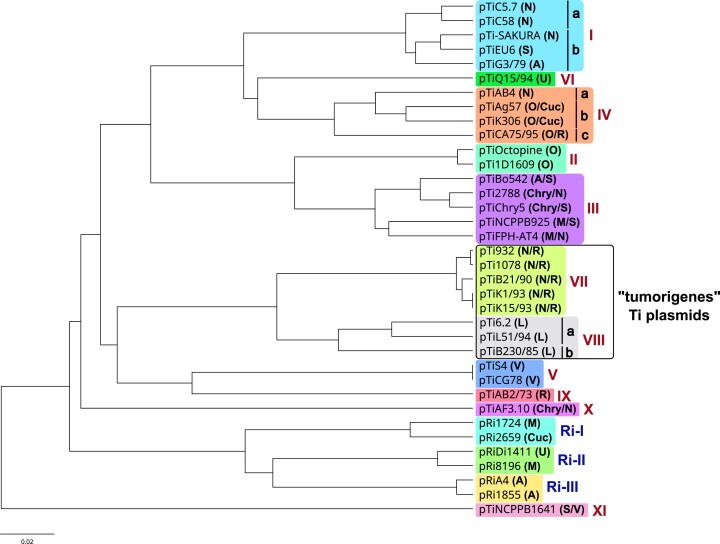
UPGMA hierarchical clustering tree based on pairwise Mash distances between Ti/Ri plasmids (supplementary table S1, Supplementary Material online). Mash sketches were produced for each plasmid (“sketch” function) with a sketch size of 10,000 and k-mer size of 15. Pairwise distances between all plasmid sequences (sketches) were calculated using the mash “dist” function in default mode. The UPGMA hierarchical clustering tree was constructed with the Python script genomic_distance_viz.py (https://github.com/laxeye/genomic-utilities). Plasmid types based on the evolutionary classification proposed by Weisberg et al. (2020, 2022) are indicated. Opine type of respective plasmid is indicated in parentheses (A, agropine; Chry, chrysopine; Cuc, cucumopine; L, leucinopine; M, mannopine; N, nopaline; O, octopine; R, ridéopine; S, succinamopine; V, vitopine; U, unknown).

Plasmids pTi932, pTi1078, and pTi6.2 associated with the “tumorigenes” clade of the family *Rhizobiaceae* were the primary subject of this study. To examine their genetic relatedness and relationships with other Ti/Ri plasmids (supplementary table S1, Supplementary Material online), we conducted Mash and AAI pairwise comparisons followed by unweighted pair group method with the arithmetic mean (UPGMA) hierarchical clustering. Both approaches allowed for accurate classification of all Ti/Ri plasmids into the types defined by Weisberg et al. (2020, 2022). Plasmids pTi932 and pTi1078 clustered with representatives of the Ti-type VII, while pTi6.2 grouped as a member of Ti-type VIII (fig. 2 and supplementary fig. S1, Supplementary Material online).

**Fig. 3.**
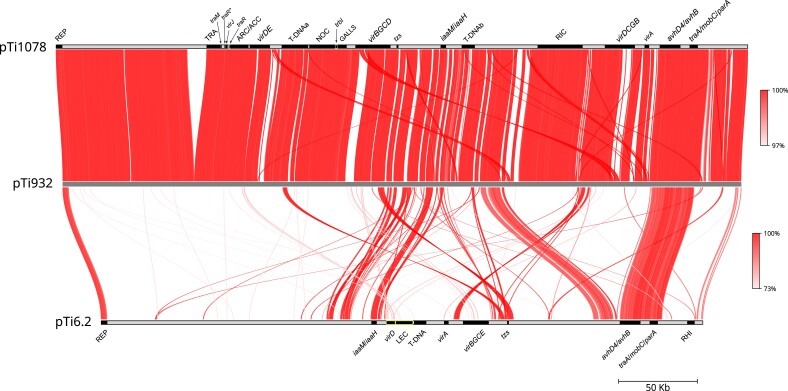
Synteny between pTi932, pTi1078, and pTi6.2. Orthologous mappings (500-bp fragment length) were computed with FastANI and plotted using the Python script visualize.py (https://github.com/moshi4/pyGenomeViz/tree/main/notebooks/fastANI). Each red line segment denotes an orthologous mapping between two replicons, indicating conserved regions. The darker color indicates a higher percentage of identity (see the legend on the right). Plasmid functional modules and specific genes are indicated as described in figure 1.

To further examine the genetic relatedness between pTi932, pTi1078, and pTi6.2, we performed average nucleotide identity (ANI) comparisons. Plasmids pTi932 and pTi1078 showed high genetic relatedness, as they exhibited 99.9% of ANI on approximately 91–94% of alignment fraction (supplementary table S2, Supplementary Material online). Accordingly, the majority of DNA regions were highly conserved among these two plasmids (fig. 3). On the other hand, pTi6.2 showed notably low conservation with pTi932 and pTi1078, as the alignment fraction ranged approximately 24–27%, although ANI between the matching regions was relatively high at approximately 93% (supplementary table S2, Supplementary Material online; fig. 3). Only backbone regions associated with plasmid replication, partitioning, and conjugation (REP, *avhD4*/*avhB* and *traA*/*mobC*/*parA*), as well as *iaaM*/*iaaH* and *tzs* genes, were mapped between pTi6.2 and pTi932/pTi1078 (fig. 3).

Mash- and AAI-distance clustering indicated genetic relationships between plasmids pTi6.2 and pTi932/pTi1078, and Ti plasmids occurring in *R. rhododendri* strains isolated from plants grown in the United States. Further comparative analysis showed high ANI (99.5–99.7%) between pTi932/pTi1078 and plasmids pTiB21/90, pTiK1/93, and pTiK15/93, on a relatively high (∼78–82%) alignment fraction (supplementary table S2, Supplementary Material online). Moreover, their backbone and pathogenicity-associated accessory gene regions were highly conserved (supplementary fig. S2*a*, Supplementary Material online). The plasmid pTi6.2 showed homology to pTiL51/94, which shared 94.6% ANI on 40% alignment fraction (supplementary table S2, Supplementary Material online). Most of their functional modules were conserved, although large DNA regions specific for each of these two plasmids were evident (supplementary fig. S2*b*, Supplementary Material online). Plasmid pTiB230/85, which belongs to the VIIIb Ti plasmid subtype related to the VIIIa Ti plasmid subtype that contains plasmids pTi6.2 and pTiL51/94 (Weisberg et al. 2022), showed poor conservation with pTi6.2 (supplementary table S2 and fig. S2*b*, Supplementary Material online). The alignment fraction between plasmids pTi6.2 and pTiB230/85 was only 22.3%, although they shared 94.6% ANI among conserved regions (supplementary table S2, Supplementary Material online). However, most of the accessory regions associated with pathogenicity are conserved between these two plasmids (supplementary fig. S2*b*, Supplementary Material online). Taken together, we will collectively refer to plasmids pTi932, pTi1078, pTiB21/90, pTiK1/93, and pTiK15/93 as type VII Ti plasmids. For plasmids pTi6.2, pTiL51/94, and pTiB230/85, the collective term “type VIII Ti plasmids” will be used.

Plasmids pTi932, pTi1078, and pTi6.2 show poor conservation with other Ti/Ri plasmids that are carried by bacteria outside of the “tumorigenes” group of *Rhizobium* spp. (supplementary Table S2 and fig. S3, Supplementary Material online). Nevertheless, we could observe some DNA stretches mapped between pTi932/pTi1078 and several other representative Ti/Ri plasmids (supplementary fig. S3*a* and *b*, Supplementary Material online). For instance, a DNA stretch encompassing ARC/ACC, *virDE*, and a part of T-DNAa of pTi932/pTi1078 shows a high degree of nucleotide identity with the corresponding regions of octopine-/ridéopine-type Ti plasmid pTiCA75/95 (supplementary figs. S3*a*, S3*b*, and S4*a*, Supplementary Material online). Furthermore, a part of the T-DNAa and NOC region is highly conserved between pTi932/pTi1078 and pTiC5.7, but also to a bit lower extent between other nopaline-type Ti plasmids (pTiC58 and pTi-SAKURA) (supplementary figs. S3*a*, S3*b*, and S4*a*, Supplementary Material online). The *virBGCD* region, including *vir* gene GALLS, of pTi932/pTi1078 shared a high degree of homology with the corresponding regions of agropine-type Ri plasmid pRi1855 (supplementary figs. S3*a*, S3*b*, and S4*b*, Supplementary Material online). Although their opine catabolism regions (RIC) showed some homology, plasmids pTi932/pTi1078 and the atypical mega Ti plasmid pTiAB2/73 were unrelated (supplementary figs. S3*a*, S3*b*, and S4*b*, Supplementary Material online).

### Backbone Regions

#### Replication and Partitioning System

Plasmids pTi6.2, pTi932, and pTi1078 carry a single putative *repABC* operon (REP), involved in plasmid replication and partitioning. The REP region was highly conserved between pTi6.2, pTi932, and pTi1078 (fig. 3), as well as with plasmids pTiB21/90, pTiK1/93, and pTiK15/93 (supplementary fig. S2*a*, Supplementary Material online). On the other hand, more divergent REP regions were found in plasmids pTiB230/85 and pTiL51/94, although the *repC* gene of the latter plasmid showed high nucleotide identity (>95.9%) and close phylogenetic relationship to the corresponding gene of type VII Ti plasmids and pTi6.2 (supplementary figs. S2*b*, S3, and S5*c*, Supplementary Material online). REP genes of the Ti plasmids of the “tumorigenes” clade do not show close evolutionary relationship to the corresponding genes of other Ti and Ri plasmids characterized so far (supplementary fig. S5, Supplementary Material online). The only exception was plasmid pTiB230/85, which clustered with pTiS4 of *Allorhizobium ampelinum* S4^T^ (supplementary table S1, Supplementary Material online) on the RepA, RepB, and RepC phylogenetic trees (supplementary fig. S5, Supplementary Material online).

#### Conjugation Systems

Plasmids pTi6.2, pTi932, and pTi1078 harbored a complete set of genes required for conjugative transfer: the *traA*/*mobC*/*parA* and *avhD4*/*avhB* genes (fig. 1). Based on gene content and organization, the conjugative transfer-associated system of plasmids pTi932, pTi1078, and pTi6.2 could be classified as belonging to the type IV transfer system (Giusti et al. 2012; Ding et al. 2013). The *avhD4*/*avhB* and *traA*/*mobC*/*parA* regions were conserved within all “tumorigenes” Ti plasmids, except for pTiB230/85. This plasmid carries a conjugation-gene cluster that resembled the gene content and organization (*tra* and *trb* operons) of conjugative transfer system of previously described Ti plasmids and could therefore be classified as belonging to the type I conjugation system, which is regulated by a QS mechanism (Ding and Hynes 2009; Ding et al. 2013). Interestingly, the type VII Ti plasmids analyzed in this study carried an additional, but incomplete, set of type I-like conjugative transfer genes. In particular, they carried *tra* genes (TRA region), including the QS regulatory genes *traM* and *traR* (fig. 1*a* and supplementary fig. S2*a*, Supplementary Material online). The *traI* gene involved in QS regulation of conjugation was absent, as were almost all *trb* genes, except for the putative gene *trbI*. Therefore, this second conjugation system in the type VII Ti plasmids is likely nonfunctional or is complemented by another AvhB/Trb system of the same (*avhD4*/*avhB* genes) or a separate coresident plasmid.

Based on the phylogenetic analysis of conjugative relaxase (TraA) protein sequences, all plasmids of the “tumorigenes” clade, except for the pTiB230/85, were classified into the MOB_P0_ group (Garcillán-Barcia et al. 2009), characterized by plasmids having a type IV transfer system (supplementary fig. S6*a*, Supplementary Material online). Together with some other alphaproteobacterial plasmids, they formed a separate new clade (named here as clade IVc) within the MOP_P0_ group. On the other hand, the conjugative transfer system of pTiB230/85 was classified into the MOB family MOB_Q2_ and clustered with other Ti plasmids included in the analysis (supplementary fig. S6*a*, Supplementary Material online). The putative TraA relaxases of the second TRA region (but lacking *trb* genes) found in type VII Ti plasmids were also classified into the MOB family MOB_Q2_. Based on the AvhB4/TrbE phylogeny (supplementary fig. S6*b*, Supplementary Material online), all plasmids were classified into the MPF_T_ group (Smillie et al. 2010). The MOB groups described above were also clearly differentiated on the AvhB4/TrbE phylogenetic tree (supplementary fig. S6*b*, Supplementary Material online).

### Accessory Regions

#### T-DNA

**Fig. 4.**
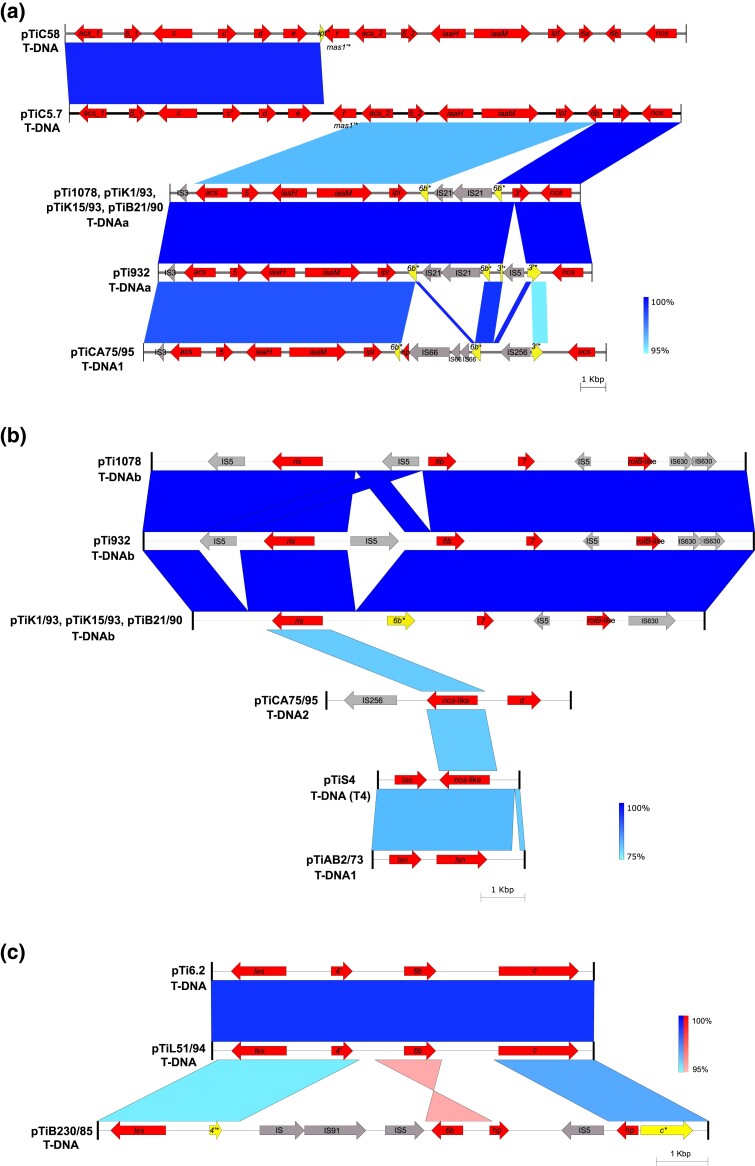
Comparative analysis of T-DNAa (*a*) and T-DNAb (*b*) of type VII Ti plasmids, and T-DNA of type VIII Ti plasmids (*c*), including related T-DNA structures. The BlastN sequence comparison (*E* value of Blast hits of 0.001 and minimum length of Blast hits of 50 bp) was performed and visualized with Easyfig. Vertical blocks indicate the identity between regions: matches to the + strand (i.e., +/+) are in blue, and matches to the − strand (i.e., +/−) are in red. The darker color indicates a higher percentage of identity (see the legend on the right). The colored arrows represent coding sequences (CDSs): intact genes (red arrows), interrupted genes (yellow arrows), and IS elements (gray arrows). Yellow rectangles represent nonfunctional gene fragments. Gene names are indicated inside the arrows. The name hp corresponds to genes encoding hypothetical proteins. Gene names with asterisks indicate interrupted genes.

Plasmids pTi932 and pTi1078 carried two T-DNAs (T-DNAa and T-DNAb) (fig. 1*a*). T-DNAa contained genes putatively encoding for the synthesis of agrocinopine and nopaline, similar to T-DNA of typical nopaline-type Ti plasmids (e.g., pTiC58). T-DNAa of pTi932 and pTi1078, and the related plasmids pTiB21/90, pTiK1/93, and pTiK15/93 of the Ti-type VII, was highly conserved (supplementary fig. S2*a*, Supplementary Material online), although some slight differences were observed (fig. 4*a*). In particular, in pTi932, unlike the other four Ti plasmids, the putative gene 3′ was interrupted by insertion element (IE) of IS5 family (fig. 4*a*).

Except for the IEs, T-DNAa of plasmids pTi932 and pTi1078 was organized similarly to that of the right side of the T-DNA of the nopaline-type Ti plasmid pTiC5.7 (genes *acs* to *nos*) (fig. 4*a*), characterized in our previous study (Kuzmanović and Puławska 2019). It was also evident that the right end of T-DNAa of the “tumorigenes” Ti plasmids (genes *6b*-*3′*-*nos*) had a higher degree of sequence identity with the corresponding T-DNA region of pTiC5.7 compared with the rest of the T-DNAa sequence (fig. 4*a*). Therefore, we further compared these T-DNAs in order to identify putative recombination breakpoints. Because syntenic blocks based on pairwise sequence-alignments generated using BlastN algorithm (Easyfig) (fig. 4) can have uneven distribution of genetic diversity along the alignment, we generated synteny plots relying on the alignment-free pairwise FastANI comparison, by setting the fragment length to 100 bp. Indeed, the synteny plot identified the location of putative recombination breakpoint within gene *5* (supplementary fig. S7, Supplementary Material online). In this respect, all the nucleotide diversity within gene *5* was located between coordinates 1 and 246, whereas the sequence from nucleotides 247–684 was almost identical (only one SNP) between pTi932/pTi1078 and pTiC5.7. Furthermore, T-DNAa of the type VII Ti plasmids largely resembles the organization of T-DNA1 of the plasmid pTiCA75/95. However, unlike T-DNAa that carries the nopaline synthesis (*nos*) gene, T-DNA1 of the plasmid pTiCA75/95 harbors a putative octopine synthesis (*ocs*) gene (fig. 4*a*). IEs interrupting genes *6b* and *3′* also differed between these two T-DNA variants (fig. 4*a*). According to the classification scheme proposed recently by Otten (2021 ), the T-DNAa of the type VII Ti plasmids and T-DNA1 of the type IVc Ti plasmid pTiCA75/95 belong to group IIb4.

The second T-DNA (T-DNAb) of pTi932/pTi1078, and the related plasmids pTiB21/90, pTiK1/93, and pTiK15/93, has an atypical gene organization and exhibited more notable variations compared with those found in their T-DNAa (fig. 4*b*). The T-DNAb structures of this group of plasmids have different IEs content, and they contain highly conserved genes putatively coding for opine synthase (*ris*) and for two putative *plast* genes (*7* and *rolB*-like) (fig. 4*b*). The T-DNAb of pT932/pTi1078 also includes the *plast* gene *6b*, whereas this gene is truncated in pTiB21/90, pTiK1/93, and pTiK15/93, although adjacent IEs could not be detected. The putative opine synthesis gene (*ris*) showed homology to opine synthesis genes of pTiCA75/95 (*nos*-like, QTG10227.1), pTiS4 (*nos*-like, ACM39650.1), and pTiAB2/73 (*lsn*, UEQ85428.1) (fig. 4*b*). The T-DNAb of type VII Ti plasmids could not be classified within any of the T-DNA groups defined by Otten (2021).

Plasmid pTi6.2 carried a single T-DNA, adjacent to a putative opine catabolic region (LEC) (fig. 1*b*). This T-DNA variant was composed of genes *les*, *4′*, and *6b* and *c* and showed a high degree of sequence identity to the T-DNA of pTiL51/94 (fig. 4*c*). On the other hand, the T-DNA of pTiB230/85 was characterized by the presence of several IEs and interrupted genes *4′* and *c*, while the gene *6b* was inverted (fig. 4*c*). The T-DNA of all type VIII Ti plasmids belong to the group IIIb of Otten (2021).

Furthermore, we performed phylogenetic analysis based on opine synthesis (*os*) genes and included additional reference plasmids. In accordance with the synteny analysis (see above and fig. 4*b*), the opine synthase of T-DNAb (*ris*) of the type VII Ti plasmids was closely related to *os* genes of pTiCA75/95 (*nos*-like, T-DNA2), pTiS4 (*nos*-like, T-DNA [T4]), and pTiAB2/73 (*lsn*- or *nos*-like, T-DNA1) (supplementary fig. S8, Supplementary Material online). They formed a well-supported clade, suggesting that they might encode for the synthesis of the same putative opine. On the other hand, the gene *les* associated with plasmids pTi6.2, pTiL51/94, and pTiB230/85 grouped separately from all other *os* genes included in the analysis, with a neighboring branch comprising L,L-succinamopine synthase (*sus*) of pTiEU6 (supplementary fig. S8, Supplementary Material online).

#### *vir* Region

The *vir* genes are unusually organized in plasmids pTi932/pTi1078 and related plasmids and are characterized by the presence of *virDE*, *virBGCD*, and *virDCGB* loci separated by other functional regions (figs. 1a and 3 and supplementary figs. S2a and S9*a*, Supplementary Material online). Furthermore, these plasmids carry GALLS, *virA*, and *virJ* genes (fig. 1*a*). The *vir* regions and genes are well conserved within this group of plasmids, although some differences in the presence or interruption of the *virD* genes were observed (supplementary fig. S9*a*, Supplementary Material online). When compared with each other, regions *virBGCD* and *virDCGB* of pTi932/pTi1078 shared approximately 89% nucleotide identity and showed similar gene organization, although they differed in orientation. In the region *virBGCD*, almost all genes were intact, except for the *virD5* gene that was interrupted by an IS5 family element (supplementary fig. S9*a*, Supplementary Material online). As the *virD5* gene product is required for efficient infection (Wang et al. 2018), the intact copy of this gene in region *virDE* probably complements its function. In phylogenetic trees based on the VirA, VirB4, VirD2, and GALLS proteins, plasmids pTi932, pTi1078, pTiB21/90, pTiK1/93, and pTiK15/93 were intertwined with other reference Ti/Ri plasmids (supplementary fig. S10, Supplementary Material online). More precisely, phylogenetic analysis suggested a close evolutionary relationship between type VII Ti plasmids and pRi1855, which relies on VirB4/VirD2 (encompassing *virBGCD* region) and GALLS proteins.

The *vir* region of pTi6.2 is also not organized as a single operon but is divided into several gene clusters (*virA*, *virD*, and *virBGCE*) (figs. 1b and 3), with the *vir* genes separated by the T-DNA and LEC regions. The *vir* genes of the related plasmids pTiL51/94 and pTiB230/85 have a similar organization as those in pTi6.2 and were highly conserved within this group of Ti plasmids (supplementary figs. S2b and S9*b*, Supplementary Material online), with the exception that *virA* is absent in pTiB230/85 (Weisberg et al. 2022) (supplementary fig. S2b, Supplementary Material online). Despite the lack of a *virA* gene in pTiB230/85 and interruption of T-DNA genes *4′* and *c* (see above), the strain B230/85 was still tumorigenic (Moore et al. 1997). Taken together, *vir* gene organization and sequence in pTi6.2, pTiL51/94, and pTiB230/85 differed from other Ti/Ri plasmid representatives (supplementary table S1, Supplementary Material online). In phylogenetic trees based on VirA, VirB4, and VirD2 protein sequences, these plasmids clustered separately from all other Ti/Ri plasmids included in this analysis (supplementary fig. S10, Supplementary Material online).

#### Opine Catabolism Regions

Plasmids pTi932/pTi1078 carried three separate regions putatively associated with catabolism of the opines agrocinopine (ACC), nopaline (NOC), and ridéopine (RIC), which were also highly conserved in plasmids pTiB21/90, pTiK1/93, and pTiK15/93 (supplementary fig. S2*a*, Supplementary Material online). The ACC region was directly linked to the ARC region associated with agrocinopine regulation of conjugation, and ARC and ACC were therefore analyzed together. The ARC/ACC region showed the same organization, with high nucleotide identity (99%), as the corresponding region of pTiCA75/95 (supplementary fig. S11*a*, Supplementary Material online). They also showed similar organization, although lower nucleotide identity (∼80%), to the ARC/ACC region of nopaline-type Ti plasmids (pTiC58, pTi-SAKURA, and pTiC5.7/pTiC6.5), and the succinamopine-type Ti plasmid pTiEU6 (supplementary fig. S11*a*, Supplementary Material online). The NOC region of pTi932/pTi1078 resembled the organization of the NOC region of nopaline-type Ti plasmids (supplementary fig. S11*b*, Supplementary Material online), sharing relatively high nucleotide identity ranging, for example, from 93.3% (pTiC58) to 96.6% (pTiC5.7).

The third putative opine-catabolic region RIC was located between the T-DNAb and *virDCGB* regions of pTi932/pTi1078 (figs. 1*a*, and 3). Similar regions were also present in pTiAB2/73, pTiCA75/95, and pTiS4 (supplementary fig. S11*c*, Supplementary Material online). As previously hypothesized (Hooykaas and Hooykaas 2021a, 2021b), this region is most likely associated with the catabolism of the opine ridéopine [*N*-(4′-aminobutyl)-d-glutamic acid], which is a condensation product of α-ketoglutarate and putrescine (Chilton et al. 2001). However, compared with the three other plasmids used for comparison, genes encoding lactamase/hydantoinase and several ABC transporter components were absent in pTi932/pTi1078 (supplementary fig. S11*c*, Supplementary Material online). We could not confidentially identify genes involved in the degradation of ridéopine, although putative genes encoding FAD-binding oxidoreductase (Rt932_00294/Rt1078) and *N*-formylglutamate deformylase (Rt932_00293/Rt1078) might be involved in this metabolic process. On the other hand, we identified putative genes (e.g., *spuC* and *gabD*) involved in degradation of ridéopine derivatives, such as putrescine and 4-aminobutanoate (GABA).

In plasmid pTi6.2, a putative gene cluster LEC associated with opine catabolism is located between the right border of the T-DNA and the *virD* operon (fig. 1b). It carries genes putatively associated with opine transport (*lecA*, *lecB*, *lecC*, and *lecD*) and metabolism (*odh*, *sacE*, *sacF*, and *sacG*), as well as *lecR* that encodes a transcriptional regulator. The LEC region was highly conserved within pTi6.2, pTiL51/94, and pTiB230/85 (supplementary figs. S2*b*and S11*d*, Supplementary Material online). This gene cluster showed similar organization but relatively low nucleotide identity (∼76%, for 63% query coverage) with the corresponding region of pTiEU6 associated with catabolism of D,L-succinamopine (supplementary fig. S11*d*, Supplementary Material online). The regions associated with catabolism of L,L-succinamopine and L,L-leucinopine in plasmids pTiBo542 and pTiChry5 differed in their sequence and organization (supplementary fig. S11*d*, Supplementary Material online).

#### Other Accessory Regions

Plasmids pTi932/pTi1078 and pTi6.2 carried large accessory regions encoding various putative and hypothetical proteins, as well as numerous insertion sequences (ISs) (fig. 1). Except for the closely related plasmids pTiL51/94, pTiB230/85, pTiB21/90, pTiK1/93, and pTiK15/93, these accessory regions were generally not conserved among the representative Ti/Ri plasmids used for comparison (supplementary table S1 and figs. S2 and S3, Supplementary Material online).

We identified putative *iaaH* and *iaaM* genes for biosynthesis of indole acetic acid (auxin) in all “tumorigenes” Ti plasmids that did not seem to be a part of a T-DNA region (fig. 1). Nonetheless, we identified a potential left T-DNA border sequence immediately upstream of the *iaaM* gene, with the coordinates 243,648–243,672 in pTi1078, and 170,901–170,925 in pTi6.2. Phylogenetic analysis of the amino acid sequences of these apparently non-T-DNA-encoded IaaH/IaaM proteins of “tumorigenes” Ti plasmids showed that they are distantly related to the IaaH/IaaM proteins commonly encoded in the T-DNA of Ti/Ri plasmids (supplementary fig. S12*a* and *b*, Supplementary Material online) and that they are instead more closely related to proteins encoded by other distantly related bacteria. Plasmids pTi6.2 and pTi932/pTi1078 contained another plant hormone (cytokinin) biosynthesis *tzs* (*trans*-zeatin synthesizing) gene, which is homologous to the T-DNA encoded *ipt* gene (fig. 1). This gene was also present in pTiL51/94 but absent from pTiB230/85, pTiB21/90, pTiK1/93, and pTiK15/93. In a Tzs protein-based phylogenetic tree, pTi6.2, pTiL51/94, pTi932, and pTi1078 clustered together and showed phylogenetic relatedness to the corresponding proteins of plasmids pTiAB2/73, pRi1724, and pRi2659, which were located on a neighboring branch (supplementary fig. S12*c*, Supplementary Material online).

Surprisingly, unlike other “tumorigenes” Ti plasmids, pTi6.2 carried the genes *rhiR*/*rhiI* involved in QS, as well as the *rhiABC* genes (RHI region). These genes were originally identified in symbiotic (Sym) plasmid pRL1JI of *Rhizobium leguminosarum* bv. *viciae*, and it has been postulated that the *rhiR*/*rhiI* QS system might be involved in nodulation efficiency (Cubo et al. 1992; Rodelas et al. 1999).

### Opine Content of Tumors Induced by Strains of the “Tumorigenes” Clade and Opine Utilization Assay

The high-performance liquid chromatography and tandem mass spectrometry (HPLC-MS^2^) analysis indicated the presence of nopaline (m/z([M + H]^+^): 305.1461) and ridéopine (m/z([M + H]^+^): 219.1345) in sunflower tumors induced by *R. tumorigenes* strains 932 and 1078^T^. Nopaline was highly abundant (min. 47 and 35× more abundant than ridéopine) in tumors induced by both strains, whereas only minor amounts of ridéopine were found (supplementary table S3, Supplementary Material online). Small amounts of ridéopine, as well as vitopine (syn. heliopine) were detected in tumors induced by a reference strain *All. ampelinum* S4^T^. Additionally, the presence of agrocinopine A could be confirmed in tumors induced by strains 932 and 1078^T^ (supplementary table S3, Supplementary Material online).

Furthermore, HPLC-MS^2^ analysis identified the presence of the iminodiacid opine leucinopine in tomato tumors induced by the strain rho-6.2^T^, based on its exact mass (m/z([M + H]^+^): 262.1285) and its fragmentation pattern (supplementary table S3 and fig. S13, Supplementary Material online). This opine was also detected in tomato tumors induced by reference strains *Agrobacterium tumefaciens* Bo542 and Chry5 that were reported to be associated with production of L,L-leucinopine (Chilton, Hood, and Chilton 1985; Chilton et al. 1995; Vaudequin-Dransart et al. 1995; Shao et al. 2018). Although leucinopine compounds detected in rho-6.2^T^, and reference strains Bo542 and Chry5 had the same exact mass, leucinopine detected in tumors caused by rho-6.2^T^ had a different retention time but highly similar MS^2^ spectra (supplementary fig. S13, Supplementary Material online). This suggests that this opine was likely an isomer of L,L-leucinopine. Phylogenetic analysis also suggested that the opine synthase encoded on pTi6.2 was distantly related to a gene associated with synthesis of L,L-leucinopine in strains Bo542 and Chry5 (supplementary fig. S8, Supplementary Material online). On the other hand, the opine synthase encoded on pTi6.2 was more similar to the succinamopine synthase of pTiEU6. Considering that strain *Agrobacterium rubi* EU6 induced the production of the D,L-form of succinamopine in plant tumors (Chilton et al. 1984; Chilton, Hood, Rinehart, and Chilton 1985), it is likely that *R. rhododendri* rho-6.2^T^ induces production of D,L-leucinopine in infected plants.

In the opine utilization assay using extracts from tumors induced by *R. rhododendri* rho-6.2^T^, *R. rhododendri* rho-6.2^T^ fully consumed leucinopine from the medium after 48 h of cultivation, as this compound could not be detected using HPLC-MS^2^ analysis at this time point (supplementary table S4, Supplementary Material online). As expected, leucinopine was also not detectable in 2× AT minimal medium mixed with sterile distilled water (ratio 1:2). On the other hand, leucinopine remained present in media inoculated with strains *A. tumefaciens* Bo542 and Chry5 (supplementary table S4, Supplementary Material online), despite robust growth of both strains. These results further confirm that the leucinopine produced by *R. rhododendri* rho-6.2^T^ differs from the leucinopine produced by *A. tumefaciens* Bo542 and Chry5.

## Discussion

### Classification of Ti Plasmids

In this study, a comprehensive comparative sequence analysis of Ti plasmids occurring in members of the clade “tumorigenes” of the family *Rhizobiaceae* (“tumorigenes” Ti plasmids) was performed. This not only primarily included plasmids pTi932, pTi1078, and pTi6.2 sequenced in our recent work (Kuzmanović et al. 2023) and reannotated in this study but also closely related plasmids associated with “tumorigenes” strains isolated in the United States (pTiK1/93, pTiK15/93, pTiB21/90, pTiL51/94, and pTiB230/85; table 1). These plasmids vary in size (∼197–455 kb) and all belong to the *repABC* plasmid family, which is typical for Ti/Ri plasmids (Farrand 1998; Pinto et al. 2012). Weisberg et al. (2020, 2022) recently reported a classification scheme that classifies Ti and Ri plasmids into 11 and three distinct types, respectively. Here, we showed that classification scheme can be accurately reproduced using a simple clustering approach based on pairwise Mash or AAI distances. For the Mash-distance-based clustering, we have prepared a pipeline that facilitates grouping of Ti/Ri plasmids, available through GitHub.

Weisberg et al. (2022) not only analyzed “tumorigenes” Ti plasmids associated with *R. rhododendri* strains isolated in the United States but also included contigs corresponding to pTi932, pTi1078, and pTi6.2 obtained in our previous studies (Kuzmanović et al. 2018; Kuzmanović et al. 2019). By analyzing the complete sequences of the latter three plasmids in this study, we were able to confirm the classification of pTi932, pTi1078, and pTi6.2 that was reported by Weisberg et al. (2022). The “tumorigenes” Ti plasmids were grouped into two types (VII and VIII). Group VII included plasmids pTi932, pTi1078, pTiK1/93, pTiK15/93, and pTiB21/90, while group VIII comprised plasmids pTi6.2, pTiL51/94, and pTiB230/85. It was also evident on the UPGMA clustering trees that “tumorigenes” Ti plasmids differ from all other Ti plasmid types included in our study (fig. 2 and supplementary fig. S1, Supplementary Material online).

We therefore conducted comprehensive analyses of “tumorigenes” Ti plasmids and their functional modules (backbone and accessory) using comparative and phylogenetic approaches. Overall, our results showed that, first, “tumorigenes” Ti plasmids have novel opine signatures compared with other Ti/Ri plasmids characterized so far, which was confirmed by tandem mass spectrometry analysis. Second, except for pTiB230/85, these plasmids carry putative conjugative transfer genes that are atypical for Ti/Ri plasmids. Third, although they have a common ancestor, “tumorigenes” Ti plasmids recombined with single or multiple distinct Ti plasmids during their evolutionary history. In this respect, our results suggest that the T-DNAa of plasmids belonging to type VII is chimera resulting from recombination between T-DNA regions of different plasmids. Each of these aspects is discussed below in more detail.

### Novel Opine Types of Ti Plasmids

Classification based on opine markers does not necessarily reflect the structural and evolutionary relatedness of Ti/Ri plasmids and therefore has limited significance in classification of these replicons. In this respect, two plasmids can belong to the same opine type despite being distantly related. For instance, plasmids pTi2788 and pTiAF3.10 are both classified as chrysopine/nopaline-type (Vaudequin-Dransart et al. 1995) but form separate lineages on our AAI and Mash distance-based trees (fig. 2 and supplementary fig. S1, Supplementary Material online) and belong to separate Ti types (supplementary table S1, Supplementary Material online). On the other hand, plasmids pTi-SAKURA and pTiEU6 that belong to nopaline- and succinamopine-type, respectively, are closely related. It was previously suggested that pTiEU6 is derived from a pTi-SAKURA-like plasmid, in which the nopaline catabolic region and the right part of the T-DNA region containing nopaline synthesis gene were replaced by the corresponding fragment of an unknown succinamopine plasmid (Shao et al. 2019). Nevertheless, because opines play an important role in the lifestyle of agrobacteria (Dessaux et al. 1998; Dessaux and Faure 2018a), we consider it important to identify which opines are associated with a particular agrobacterial strain or its Ti/Ri plasmid and thus obtain a more complete overview of the characteristics of Ti/Ri plasmids.

Bioinformatic analysis indicated that the T-DNAa of type VII Ti plasmids encodes production of agrocinopine and nopaline. The presence of these two opines in plant tumors induced by the representative strain 1078^T^ was confirmed by HPLC-MS^2^ analysis. The T-DNAb of type VII Ti plasmids also carries the *ris* gene that putatively encodes an opine synthase. Synteny and phylogenetic analyses suggested the *ris* gene is related to the opine synthesis genes of the well-characterized plasmids pTiS4 (*nos*-like) and pTiAB2/73 (*lsn*). *All. ampelinum* S4^T^ induces production of opines vitopine and ridéopine in infected plants (Szegedi et al. 1988; Chilton et al. 2001). Nevertheless, the gene encoding ridéopine synthase (*ris*) was unknown, although the putative opine synthase (*nos*-like) located on the T-DNA (T4) represents a clear candidate. The characteristics of opine(s) in tumors induced by AB2/73 were not tested. However, it was recently hypothesized by Hooykaas and Hooykaas (2021a) that *lsn*- and *nos*-like genes in pTiAB2/73 and pTiS4, respectively, encode for synthesis of the opine ridéopine. Therefore, we assume that the *ris* gene of type VII Ti plasmids most likely encodes synthesis of this opine. This assumption is further supported by the chemical analysis of tumors induced by strains 932 and 1078^T^.

T-DNA of type VIII Ti plasmids (pTi6.2, pTiL51/94, and pTiB230/85) contained a putative gene *les* associated with synthesis of an unknown opine. Phylogenetic analysis performed in this study suggested that the gene *les* is most likely associated with the synthesis of a putative opine belonging to the iminodiacid subclass of opines (i.e., nopaline, octopine, leucinopine, and succinamopine). Interestingly, strain B230/85 was studied previously and shown to induce tumors containing an unknown iminodiacid opine of the succinamopine–leucinopine type (provisionally designated IDA-B) (Moore et al. 1997). Indeed, HPLC-MS^2^ analysis of tumors induced by strain rho-6.2^T^ indicated the presence of leucinopine. Unlike the L,L-leucinopine produced in tumors induced by strains Chry5 and Bo542, rho-6.2^T^-specific leucinopine most likely has D,L stereochemistry, which is consistent with the phylogenetic analysis. This is further supported by the fact that strains Bo542 and Chry5 could not efficiently degrade leucinopine originating from tumors induced by strain rho-6.2^T^ in our opine utilization assay, despite being able to utilize L,L-leucinopine (Chilton, Hood, and Chilton 1985; Chilton et al. 1995; Vaudequin-Dransart et al. 1995).

### Unusual Conjugative Transfer Systems in Ti Plasmids

The conjugal transfer systems of Ti plasmids described to date are organized into *tra* and *trb* operons and are classified as type I conjugation systems (Ding and Hynes 2009; Ding et al. 2013). The conjugation of this plasmid class is regulated by a QS system (Farrand 1998). However, with the exception of pTiB230/85, “tumorigenes” Ti plasmids have conjugative transfer system genes that are unusual for Ti plasmids. Their conjugative transfer genes are composed of *avhD4*/*avhB* and *traA*/*mobC*/*parA* regions and can be classified into the type IV conjugation system (Ding et al. 2013), within a new clade we termed IVc. Conjugal transfer of plasmids possessing a type IV conjugation system can be regulated by different genes (Giusti et al. 2012; Ding et al. 2013; Pistorio et al. 2013). Interestingly, the conjugation system of plasmid pTiAB2/73 can also be classified as type IV, but it belongs to the separate clade IVa. Furthermore, group IV includes some rhizobial Sym plasmids, as well as some non-Ti plasmids associated with agrobacteria, such as the relatively small (∼79 kb) plasmid pAtS4 carried by *All. ampelinum* S4^T^ (Ding et al. 2013). Plasmid pAtK84c (∼388 kb) of the well-known biocontrol strain *Rhizobium rhizogenes* K84 also has a type IV conjugation system. Moreover, our results indicated that the diversity of conjugal systems of Ti plasmids was not limited to types I and IV. Surprisingly, a notably large (605 kb) Ti plasmid pTiNCPPB1641 was characterized by a type II conjugation system (reviewed by Ding and Hynes 2009), which is also found in pAtC58, an accessory megaplasmid harbored by the well-known tumorigenic strain C58.

### Origin and Evolution of Ti Plasmids Associated with the Clade “tumorigenes”

The “tumorigenes” Ti plasmids can be divided into types VII and VIII (Weisberg et al. 2022). These two Ti plasmid types have different sets of accessory genes, suggesting that they have different evolutionary histories. The modularity of type VII and VIII Ti plasmids was also suggested by Weisberg et al. (2022). Possible scenarios for the evolution of these Ti plasmids are detailed below.

**Fig. 5.**
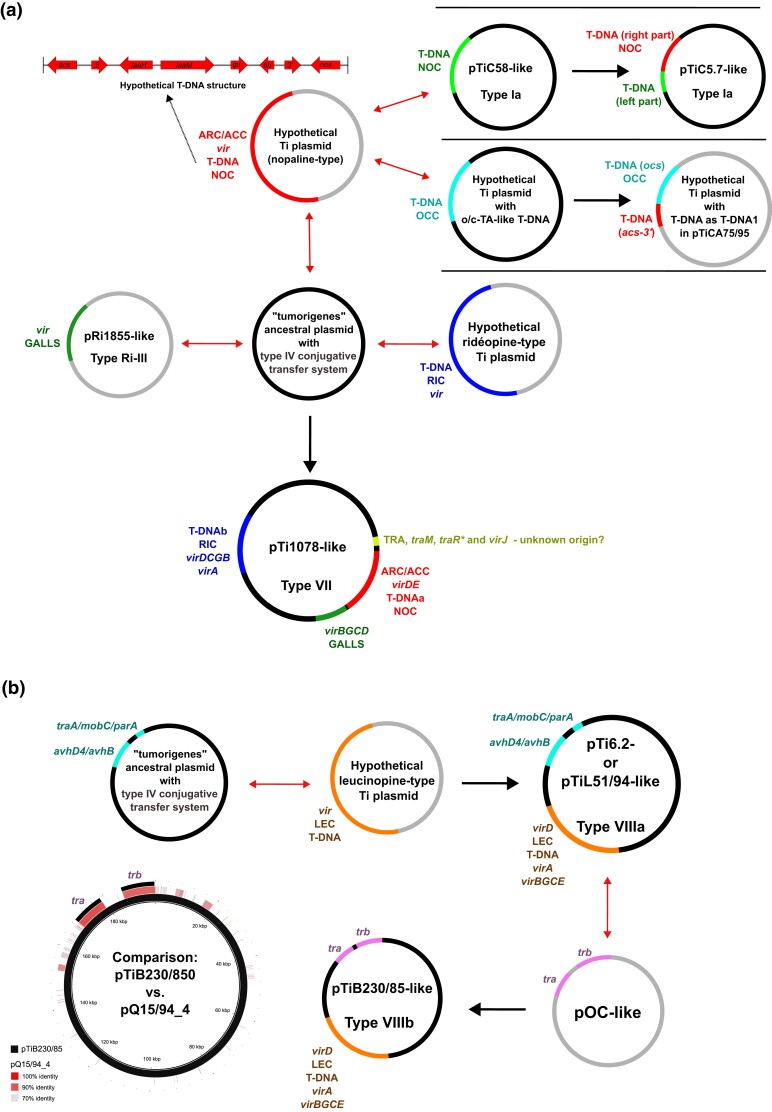
Reconstruction of the evolutionary history of “tumorigenes” Ti plasmids, including type VII (*a*) and type VIII (*b*) Ti plasmids. See Discussion section for more details.

The plasmids belonging to Ti-type VII appear to have had a complex evolutionary history. As all the plasmids of this group are highly similar, we refer here to the representative member pTi1078. In plasmid pTi1078, at least four distinct Ti/Ri plasmid-like blocks could be distinguished. These blocks appear to have been acquired through recombination (cointegration) with other plasmids, as suggested by our synteny analysis (supplementary figs. S3 and S4, Supplementary Material online). The most interesting was the block that includes regions ARC/ACC, *virDE*, T-DNAa, and NOC. The high nucleotide identity of this block to the corresponding regions of plasmids pTiC5.7 (Ti-type Ia) and pTiCA75/95 (Ti-type IVc) suggests that they have a common origin. In particular, we propose that the hypothetical ancestral nopaline-type Ti plasmid comprising this block cointegrated with the “tumorigenes” ancestral plasmid with a type IV conjugative transfer system (fig. 5). The hypothetical ancestral nopaline-type Ti plasmid most likely had an *acs-5-iaaH-iaaM-ipt-6a-3′-nos* gene combination in its T-DNA. Separate recombination events involving this hypothetical ancestral nopaline-type Ti plasmid and some other plasmids appear to have played a role in the evolution of plasmids belonging to Ti types Ia (pTiC5.7 like) and IVc (pTiCA75/95 like). In particular, a pTiC5.7-like plasmid was probably derived from the hypothetical ancestral nopaline-type Ti plasmid and a pTiC58-like plasmid (fig. 5). In this recombination event, the right side of T-DNA and adjacent NOC region of pTiC58-like plasmids was exchanged for the corresponding sequence of the ancestral Ti plasmid (nopaline-type) (fig. 5). Similarly, we propose that there was another independent recombination event, in which the right end of the T-DNA (*nos* gene) and NOC region of the hypothetical ancestral nopaline-type Ti plasmid was replaced by the octopine synthase (*ocs*) gene and octopine catabolic (OCC) region of a hypothetical Ti plasmid with o/c-TA-like T-DNA, thus generating a structure that can be found in pTiCA75/95. However, plasmid pTiCA75/95 seems to be a result of cointegration with additional plasmids (Weisberg et al. 2020). The second block comprises T-DNAb, RIC, and *virDCGB* regions, as well as a *virA* gene. As this block did not show high identity to any of the available Ti/Ri plasmid sequences, we speculate that it might originate from a hypothetical ridéopine-type Ti plasmid (fig. 5). Although bioinformatic analyses suggested that plasmids pTiAB2/73 and pTiCA75/95 also carry T-DNAs with a *ris* gene and the RIC region, synteny analysis showed that they clearly diverged during their evolution (fig. 4*b* and supplementary fig. S11*c*, Supplementary Material online). The third block encompassing the *virBGCD* region and the GALLS gene probably originated from a Ri plasmid similar to pRi1855 (fig. 5). The GALLS gene-encoded protein substitutes for the VirE2 protein and was previously found in some Ri plasmids lacking the *virE* gene (Hodges et al. 2004). The fourth block includes the TRA region, as well as the *traM* gene, two fragments of an interrupted *traR* gene, and a *virJ* gene. As this block showed relatively low similarity to other Ti/Ri plasmids included in our analysis and sequences available in GenBank, except for type VII members, it most likely originates from an unknown plasmid.

The plasmids of Ti-type VIII (pTi6.2, pTiL51/94, and pTiB230/85) appear to have a simpler evolutionary history. This plasmid group most likely resulted from a cointegration of the “tumorigenes” ancestral plasmid bearing a type IV conjugative transfer system (common ancestor with type VII Ti plasmids) with an unknown leucinopine-type Ti plasmid (fig. 5). As suggested by phylogenetic and synteny analyses (see Results section), this hypothetical leucinopine-type Ti plasmid differs notably from all Ti/Ri plasmids described so far, and we were not able to identify related plasmids through GenBank searches. Because they were located separately, it is unclear if the *virA*/*virBGCE* and *virD* regions were introduced in independent cointegration events, although there are no clear indicators for such a scenario. After cointegration with a hypothetical leucinopine-type Ti plasmid, lineages corresponding to pTi6.2, pTiL51/94, and pTiB230/85 appear to have exchanged DNA material with different replicons. For instance, apart from modules associated with the pathogenicity and conjugative transfer, other regions were less conserved between pTi6.2 and pTiL51/94. On the other hand, plasmid pTiB230/85 appears to have recombined with an unknown plasmid, because its modules for a type IV conjugative transfer system were replaced with genes for a type I conjugative transfer system (fig. 5). The Blast searches suggested that the donor strain might be a plasmid similar to the non-Ti/Ri plasmid pQ15/94_4 (accession: CP049221.1) (fig. 5).

### Evolution of T-DNA

T-DNA regions are highly diverse and highly chimeric structures. For instance, Otten (2021) identified 92 different T-DNA region types within 350 strains and classified them into three main groups. As suggested in the former study, a complete reconstruction of the evolutionary relationships between some T-DNA regions is hindered by the absence of evolutionary intermediates. By performing a thorough comparative analysis, we could gain some insights into the evolution of the T-DNA regions of the “tumorigenes” Ti plasmids and related T-DNA structures.

As indicated above, the T-DNAa of type VII Ti plasmids appears to have originated from a hypothetical T-DNA structure composed of *acs*-*5*-*iaaH*-*iaaM*-*ipt*-*6b*-*3′*-*nos* genes (fig. 5). Since the acquisition of this T-DNA variant by the common ancestor of type VII Ti plasmids, T-DNAa diverged through large-scale events, namely, by insertion of IS elements. Interestingly, the insights gained into the structure of T-DNAa facilitated elucidation of the evolution of some other T-DNA variants. In particular, in our previous study, we analyzed Ti plasmid pTiC5.7 and tried to reconstruct the evolutionary history of its T-DNA (Kuzmanović and Puławska 2019). The T-DNA of pTiC5.7 has an identical sequence as that of pTiKerr108 (accession: MK439384.1), which is classified into the group IIb3 by Otten (2021). Guided by previous studies investigating similar T-DNA structures (Drevet et al. 1994; Otten and De Ruffray 1994), we hypothesized that the T-DNA of pTiC5.7 is derived from pTiC58-like T-DNA (left side) and an unknown T-DNA (right side) and identified a probable recombination breakpoint located within gene *5* (Kuzmanović and Puławska 2019). Surprisingly, T-DNAa of the type VII Ti plasmids comprises the missing part to fully reconstruct the evolution of pTiC5.7 T-DNA. In particular, the right side of the pTiC5.7 T-DNA most likely originates from the ancestral T-DNA structure that led to T-DNAa of type VII Ti plasmids. Moreover, the T-DNA1 structure of pTiCA75/95 also seems to be derived from this ancestral T-DNA structure. In T-DNA1 of pTiCA75/95, the *nos* gene is replaced with the *ocs* gene.

T-DNAb of the type VII Ti plasmids and the single T-DNA of type VIII Ti plasmids have unusual organization. However, as closely related T-DNA structures are currently unavailable, we were unable to elucidate their evolutionary relationships with other T-DNAs. Although the putative ridéopine synthase of T-DNAb of type VII Ti plasmids showed homology to the corresponding loci found in pTiCA75/95 (T-DNA2), pTiS4 (T-DNA [T4]), and pTiAB2/73 (T-DNA1), the rest of their T-DNA was different. Possibly, these T-DNA structures have a common origin followed by a long evolutionary history leading to their divergence. It is noteworthy to point out that Otten (2021) analyzed the T-DNA structure derived from a draft genome sequence of strain 1078^T^, which only partly corresponds to the T-DNAb of pTi1078 (group IIIc) reported in this study. The one analyzed by Otten (2021) lacks the putative ridéopine synthase gene and contains an additional copy of gene *7*. We believe these inconsistencies are due to the misassembly of this T-DNA structure in Otten (2021) due to it being fragmented in several contigs, highlighting the importance of complete genome assemblies in understanding T-DNA structure and evolution. In contrast, the T-DNAb of pTi1078 reported here is derived from a complete genome sequence obtained by a hybrid sequencing approach and could be considered authentic. In the case of the type VIII Ti plasmids, their T-DNA corresponds to group IIIb, together with the T-DNA of *Allorhizobium vitis* strain CG957 (accession: WPHP00000000.1) (Otten 2021).

All eight “tumorigenes” Ti plasmids analyzed in this study carried putative *iaaH* and *iaaM* genes that appear to be outside of a T-DNA region, unlike what was reported by Weisberg et al. (2022), who analyzed complete Ti plasmids of “tumorigenes” strains isolated in the United States. Similarly, non-T-DNA *iaaH* and *iaaM* genes were also identified in pTiAB2/73 (Hooykaas and Hooykaas 2021a). Additionally, phylogenetically related *iaaH*/*iaaM* genes were reported in pTiQ15/94 (supplementary fig. S12*a* and *b*, Supplementary Material online), although it is unclear if these genes are the part of T-DNA (Weisberg et al. 2020). As “tumorigenes” Ti plasmids have little in common with pTiAB2/73 and pTiQ15/94, the origin of these genes is puzzling. These genes were phylogenetically more closely related to *iaaH*/*iaaM* genes occurring in distantly related bacteria, such *Dickeya* spp., *Paraburkholderia* spp., *Pantoea*, *Pseudomonas savastanoi* pvs., *Pectobacterium*, and *Xanthomonas arboricola* (supplementary fig. S12*a* and *b*, Supplementary Material online). These bacteria were reported as plant associated and include well-known plant pathogens. For instance, in *P. savastanoi* pv. *savastanoi*, *iaaH*/*iaaM* genes are encoded on chromosomes and their product indoleacetic acid is involved in knot (tumorous gall) development (Rodríguez-Moreno et al. 2009; Ramos et al. 2012). Although the presence of non-T-DNA *iaaH*/*iaaM* genes in Ti plasmids is uncommon, their activity may influence tumor development. In this respect, a non-T-DNA gene *tzs* that participates in cytokinin synthesis, and is also present in pTi932, pTi1078, and pTi6.2, is involved in regulation of *vir* gene expression and bacterial growth during *A. tumefaciens* infection (Hwang et al. 2013).

## Conclusion

To date, the “tumorigenes” Ti plasmids have been found only within the relatively limited phylogenetic group including only two species, *R. tumorigenes* and *R. rhododendri*. Overall, we hypothesize that these plasmids have a common origin, but they diverged through large-scale recombination events, rather than point mutations. As for other Ti plasmids, the cointegration of two Ti/Ri plasmids, or Ti/Ri plasmid and non-Ti/Ri plasmid, followed by resolution through homologous recombination and emergence of new plasmid sequence combinations seems to represent the major driving force shaping the evolution of “tumorigenes” Ti plasmids. Indeed, in vitro studies clearly showed evidences for cointegration events involving different Ti plasmids (Hooykaas et al. 1980). Likewise, a similar mechanism of generation of cointegrates and their resolution was proposed for some rhizobial plasmids (Brom et al. 2004). Moreover, our study indicated that Ti and Ri plasmids can be clearly differentiated using Mash- and AAI-distance clustering. The ease of implementing our approach will facilitate the rapid classification of new Ti/Ri plasmids as they are identified in future studies. Taken together, our study contributes to a better understanding of the evolution and diversification of Ti plasmids and opens up a number of future research questions. Additionally, this work provides a solid ground to further study the epidemiology and ecology of crown gall, as well as to improve disease control measures, particularly disease diagnostics.

## Materials and Methods

### Plasmid Sequences and Bacterial Strains

In this study, we primarily analyzed the Ti plasmid sequences of strains belonging to the “tumorigenes” clade: *R. tumorigenes* strains 932 (pTi932) and 1078^T^ (pTi1078) and *R. rhododendri* rho-6.2^T^ (pTi6.2) (table 1). Complete genome sequences of these strains were reported in our previous study describing the new species *R. rhododendri* (Kuzmanović et al. 2023). Additionally, the related plasmid sequences of *R. rhododendri* strains originating from the United States were included (table 1). Moreover, various reference Ti/Ri plasmids were included in our comparative analyses (supplementary table S1, Supplementary Material online).

The three strains of the “tumorigenes” clade mentioned above, as well as *A. tumefaciens* strains Bo542 and Chry5 and *All. ampelinum* strain S4^T^ (supplementary table S1, Supplementary Material online), were used for plant inoculation (see below). For this purpose, bacterial strains were grown on solid tryptone-yeast extract (TY) (Kuzmanović et al. 2019) or yeast mannitol agar (YMA) (Kuzmanović et al. 2015) media at 28 °C for 24–48 h.

### Annotation and Circular Plasmid Maps

Complete Ti plasmid sequences pTi932, pTi1078, and pTi6.2 were reannotated using Prokka version 1.14.5 (Seemann 2014). Furthermore, sequences of interest were functionally annotated through BlastP comparison against the NCBI nonredundant (nr) protein database (https://blast.ncbi.nlm.nih.gov/Blast.cgi; last accessed on January 2023). IS elements were identified using ISEscan version 1.7.2.3 (Xie and Tang 2017). Annotations were manually curated and deposited in Figshare depository (https://doi.org/10.6084/m9.figshare.23500758 ).

### Plasmid Sequence Comparisons

For differentiation and grouping of Ti/Ri plasmids, we prepared a pipeline relying on Mash-distance clustering, which was inspired by similar approaches for plasmid typing (Acman et al. 2020; Robertson et al. 2020; Weisberg et al. 2020). In brief, in this study, Mash sketches were produced for each plasmid (“sketch” function) with a sketch size of 10,000 and a k-mer size of 15, using Mash version 2.3 (Ondov et al. 2016). Subsequently, pairwise distances between all plasmid sequences (sketches) were computed using the Mash “dist” function in default mode. The pairwise Mash distances were then used to construct an UPGMA hierarchical clustering tree with the Python script genomic_distance_viz.py (https://github.com/laxeye/genomic-utilities). The full pipeline is available for reuse at https://github.com/diCenzo-Lab/010_2023_Agrobacterial_Ti_plasmids. Additionally, we also grouped plasmids based on AAI-distance clustering. AAI values were computed using the CompareM software (https://github.com/dparks1134/CompareM) using the aai_wf command with the BlastP option and other default parameters. The AAI distances were clustered using the UPGMA method as described above.

The more closely related plasmids were compared by means of an ANI method using FastANI version 1.2, which estimates ANI using Mashmap as its MinHash-based alignment-free sequence mapping engine (Jain et al. 2018). The fragment length was set to 500 bp. Because FastANI is designed to estimate ANI between moderately divergent sequences (i.e., in the 80–100% identity range), it only reports reciprocal mappings with alignment identities close to 80% or higher.

Circular visualization of whole-plasmid sequence comparisons were performed using Blast Ring Image Generator (BRIG) Version 0.95 (Alikhan et al. 2011). BRIG analysis involved the calculation of the percent of sequence identity and sequence coverage, with respect to a reference plasmid. The analysis was performed using the BlastN option. Moreover, to visualize synteny between whole-plasmid sequences, we exploited orthologous mappings between replicons computed using FastANI (see above). Orthologous mappings were plotted using the Python script visualize.py (https://github.com/moshi4/pyGenomeViz/tree/main/notebooks/fastANI), which is a part of pyGenomeViz genome visualization Python package (https://github.com/moshi4/pyGenomeViz).

Synteny plots between plasmid regions of interest were made using Easyfig version 2.2.5 (Sullivan et al. 2011). The BlastN option was used with a maximum *E* value of Blast hits of 0.001 and minimum length of Blast hits of 50 bp. Additionally, we also employed the pgv-fastani.py script for visualization of orthologous mappings computed using FastANI. This script is available within pyGenomeViz (see above). For this purpose, the fragment length was set to 100 bp.

The NCBI BlastN and BlastP were used for ad hoc sequence comparisons at the nucleotide and amino acid levels, respectively.

### Phylogenetic Analysis

Protein sequence alignments were generated using MAFFT version 7.5.1.5 (Katoh et al. 2019) with default parameters and then trimmed with trimAl version 1.4.rev15 (Capella-Gutiérrez et al. 2009) with the “automated1” option. Maximum likelihood (ML) phylogenies were inferred using IQ-TREE version 2.0.3 (Nguyen et al. 2015). Model selection was conducted using IQ-TREE ModelFinder (Kalyaanamoorthy et al. 2017) based on Bayesian information criterion (BIC) (Schwarz 1978). Branch supports were assessed by ultrafast bootstrap analysis (UFBoot) (Hoang et al. 2018) and the SH-aLRT test (Guindon et al. 2010) using 1,000 replicates. The trees were visualized using FigTree version 1.4.4 (https://github.com/rambaut/figtree) and edited using Inkscape version 1.2.1 (https://inkscape.org/).

### Plant Inoculation

For analysis of the opine content, plant tumor tissue was obtained by inoculating sunflower (*Helianthus annuus* L. hybrid “NK Neoma”) seedlings and young tomato plants (*Solanum lycopersicum* cv. Harzfeuer). Sunflower seedlings were inoculated with tumorigenic bacterial strains of *R. tumorigenes* (932 and 1078^T^) and *A. tumefaciens* (Bo542 and Chry5). Tomato plants were inoculated with strains of *R. rhododendri* (rho-6.2^T^) and *All. ampelinum* (S4^T^) (supplementary table S1, Supplementary Material online). The sunflower seedlings were inoculated in the hypocotyl at the stage of two true leaves by transferring a mass of 24-h-old bacteria grown on YMA with a sterile toothpick into wounds (one per plant, 1-cm incision) made aseptically with a scalpel. Seedlings were inoculated immediately before their transplanting to pots. Tomato plants were inoculated at the stage of two to three true leaves through wounds made in the stem below the cotyledons. Bacteria grown on TY medium were transferred with a sterile toothpick or pipetting 5 *μ*l of bacterial suspension (2–3 × 10^9^ colony-forming units [CFU]/mL) made in 10 mm MgCl_2_ buffer. The inoculation sites were wrapped with aluminum foil to protect the wound from drying out. Wounded but noninoculated plants were used as the negative controls. At least four replicate plants (tomato and sunflower) were inoculated per strain. The first 3–5 days after inoculation, the relative humidity was set at 90%. Plants were subsequently maintained at a temperature of 19 ± 1 °C with a 14-h light photoperiod and a humidity of approximately 70%. The sampling of tumor tissue was conducted approximately 4–5 weeks after inoculation. Fragments of fresh tumor tissue were cut with a sterile scalpel, placed in 2-ml Eppendorf tubes, frozen in liquid nitrogen, and stored at −80 °C until chemical analysis. For opine utilization assays, fresh tumors induced by *R. rhododendri* rho-6.2^T^ were processed directly after sampling (see below).

### Preparation of Opine Extracts from Tumor Tissue and Opine Utilization Assay

For identification of opines in tumor tissue, tumors on inoculated tomato and sunflower plants were analyzed. Briefly, 40–85 mg of tumor tissue of tomato plants was mixed with distilled water (10 *µ*l/mg of tissue weight) and sonicated in an ultrasonic bath at 70 °C for 15 min. Subsequently, 300-*µ*l methanol was added, briefly vortexed, and again sonicated at 70 °C for 2 min. A 500-*µ*l chloroform was added and vortexed, and after centrifugation (13,000 *g*, 5 min, 4 °C), 600 *µ*l of the polar phase was evaporated to dryness. For preparation of opine extracts from sunflower tumors, 100- to 800-mg tumor tissues were mixed with distilled water (3 *µ*l/mg of tissue weight) and boiled for 10 min at 100 °C in a water bath. The softened tissue was transferred to a 5-ml Eppendorf tube and crushed with a spatula. After vortexing briefly, the material was centrifuged at room temperature and 10,000 *g* for 10 min. The supernatants were filtered through 0.22-*µ*m cellulose acetate membrane filters and subsequently mixed with four times the volume of ice-cold acetone. After vortexing for 1 min, the suspensions were kept at −20 °C for 60 min. Afterward, samples were centrifuged at 13,000 *g* for 10 min at 4 °C and 700 *μ*l of the clear supernatant was evaporated to dryness. Both tomato and sunflower tumor extracts were stored at −80 °C until further processing.

For opine utilization assays, tumor extracts containing opines were prepared in a similar way as described before (Vaudequin-Dransart et al. 1995). Fresh tumors developed on tomato plants inoculated with the strain *R. rhododendri* rho-6.2^T^ were sliced and placed into glass Erlenmeyer flasks. Then, distilled water (3 ml/g of sample) was added, and the flask was placed in boiling water for 10 min. After cooling down to room temperature, the mixture was transferred to a mortar and the tissue was crushed with a pestle. The mixture was then transferred to a Falcon tube and vortexed vigorously. The liquid phase was filtered through cheesecloth and/or filter paper and centrifuged at 10,000 *g* for 10 min at 4 °C to remove tumor tissue debris. The supernatant was filtrated through 0.22-*µ*m cellulose acetate membrane filters and stored at −20 °C until further processing. Leucinopine utilization assays were performed in a degradation medium consisting of 2× AT minimal medium (Tempé et al. 1977; Morton and Fuqua 2012) supplemented with ammonium sulfate (2 g/L, for 2× medium), which was mixed with tumor extract in 1:2 ratio. Five milliliters of the degradation medium (three replicas) was inoculated with 100 *µ*l of bacterial suspension (∼10^8^ CFU/ml). The strains *R. rhododendri* rho-6.2^T^ and *A. tumefaciens* Bo542 and Chry5 were used. Noninoculated degradation medium and 2× AT minimal medium mixed with sterile distilled water instead of tumor extract (ratio 1:2) were used as controls. Culture tubes were incubated at 28 °C on a rotary shaker (200 rpm). A 1 ml of the supernatant was sampled after 48 h and stored at −80 °C until further processing. For extraction of leucinopine, the samples were mixed with four times the volume of ice-cold acetone and further processed as described above for supernatants derived from sunflower tumor samples. Samples were stored at −20 °C until further analysis.

### HPLC-MS^2^ of Opines from Tumor Tissue

Chromatographic separation and MS measurement were performed on an Agilent 1290 Infinity II LC-System, coupled to an Agilent 6545-Q-Tof mass spectrometer (Agilent Technologies, Waldbronn, Germany), equipped with a Gemini C18 reversed-phase analytical column (150 × 2 mm, 3-*µ*m particle size; Phenomenex, Aschaffenburg, Germany). The column was equilibrated at a ratio of buffer A (50 mm ammonium formate, pH 8.1) and buffer B (methanol) of 95:5. Dried extracts were resuspended in 100-*µ*l buffer A and analyzed at a constant temperature of 35 °C using the following gradient at a constant flow rate of 0.22 ml/min: 1 min 95% A, 19 min gradient to 70% A, 17-min gradient to 5% A, followed by a constant period at 5% A for 4 min. Column re-equilibration was achieved using a 2-min gradient to 95% A, followed by a constant period at 95% A for 4 min. MS analysis was performed in positive ion mode with a rate of 3 Hz for data acquisition and automated MS^2^ acquisition. Full scan mass spectra were recorded from 90 to 1,178 m/z. Data analysis was performed using the Mass Hunter Qualitative Analysis Software B.08.00 (Agilent Technologies). Opines were identified based on the accurate mass of their [M + H]^+^ ions and their MS^2^ fragmentation pattern (Padilla et al. 2021).

## Supplementary Material

Supplementary data are available at *Genome Biology and Evolution* online (http://www.gbe.oxfordjournals.org/).

## Supplementary Material

evad133_Supplementary_Data

## Data Availability

Annotations of plasmids pTi932, pTi1078, and pTi6.2 were deposited in Figshare depository (https://doi.org/10.6084/m9.figshare.23500758). The pipeline for grouping of Ti/Ri plasmids is available in GitHub (https://github.com/diCenzo-Lab/010_2023_Agrobacterial_Ti_plasmids). Other data underlying the results published in this article are available within its electronic Supplementary material.
