# Supplementary material for: Diversity and Evolutionary History of Ti Plasmids of “tumorigenes” Clade of Rhizobium spp. and Their Differentiation from Other Ti and Ri Plasmids
Source: Genome Biol Evol. 2023 Jul 18;15(8):evad133. doi: 10.1093/gbe/evad133 (PMC10410297; doi:10.1093/gbe/evad133)
Supplement: evad133_Supplementary_Data [file Supplementary_Figures.pdf › Supplementary_Figures.pdf]

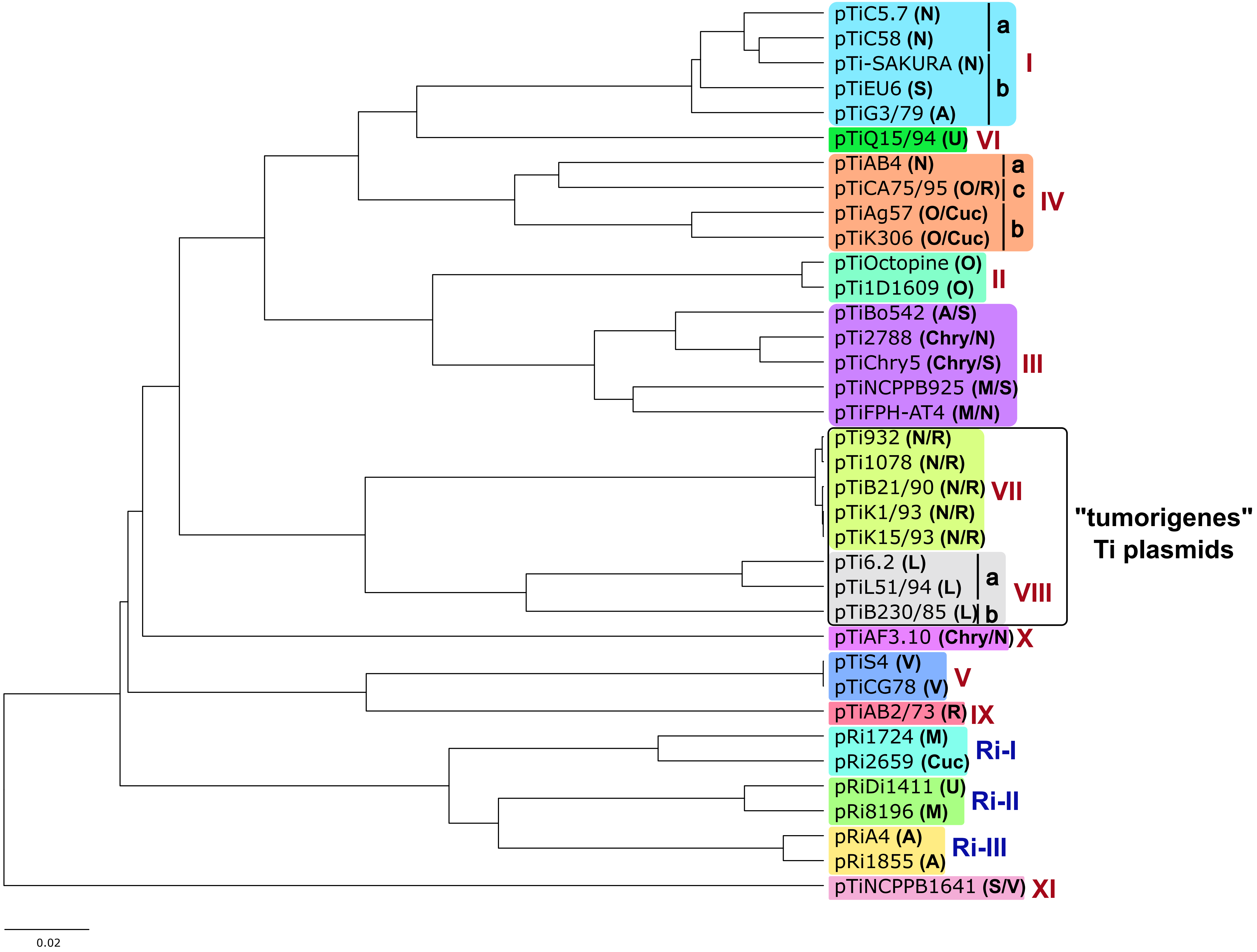

Fig. S1. Unweighted Pair Group Method with the Arithmetic mean (UPGMA) hierarchical clustering tree based on pairwise average amino acid identity (AAI) distances between Ti/Ri plasmids (Table S1). AAI values were computed using the CompareM software (github.com/dparks1134/CompareM) using the aai_wf command with blastp option, and other default parameters. The UPGMA hierarchical clustering tree was constructed with the Python script genomic_distance_viz.py (https://github.com/laxeye/genomic-utilities). Plasmid types based on the evolutionary classification proposed by Weisberg et al. (2020; 2022) are indicated.

a.

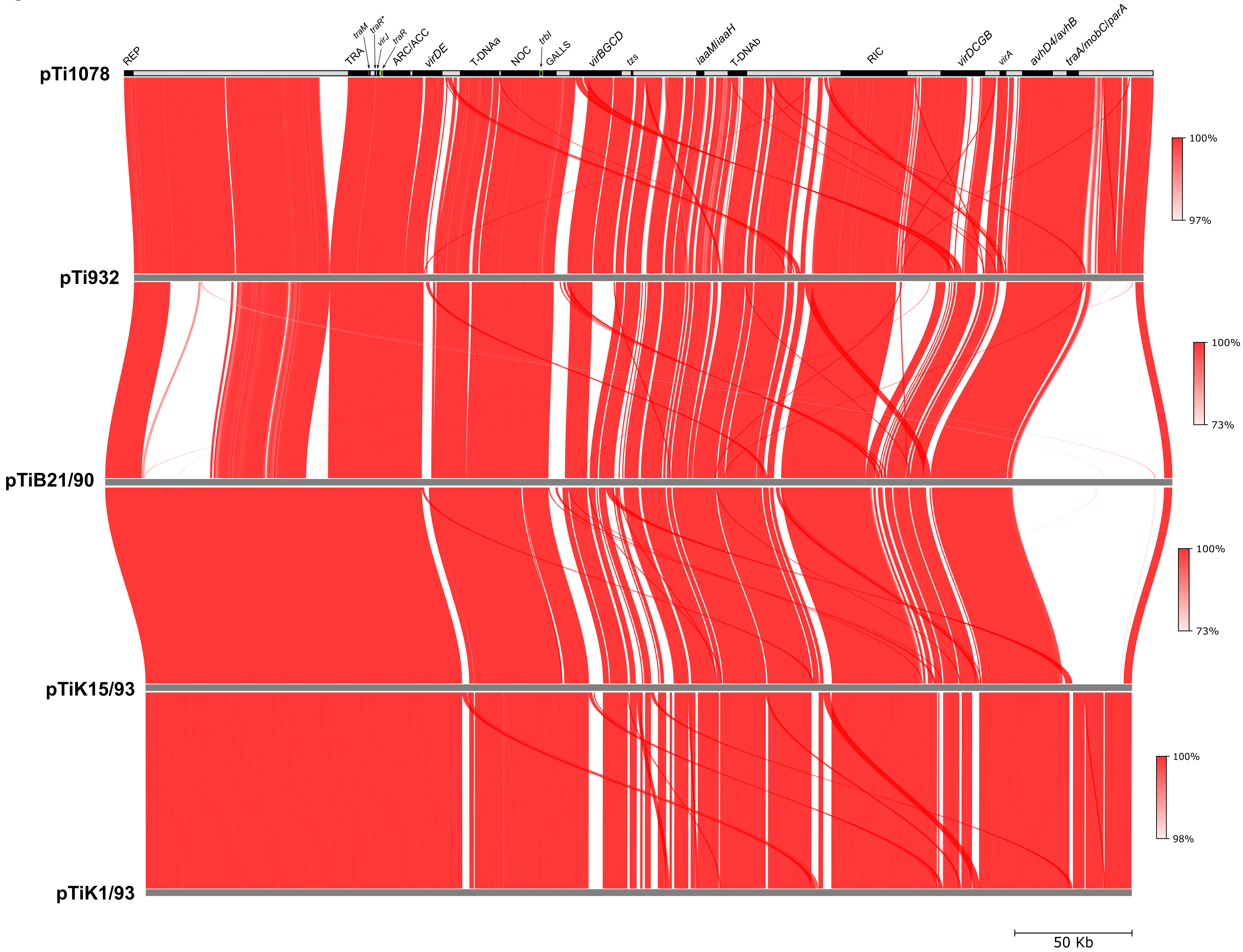

b.

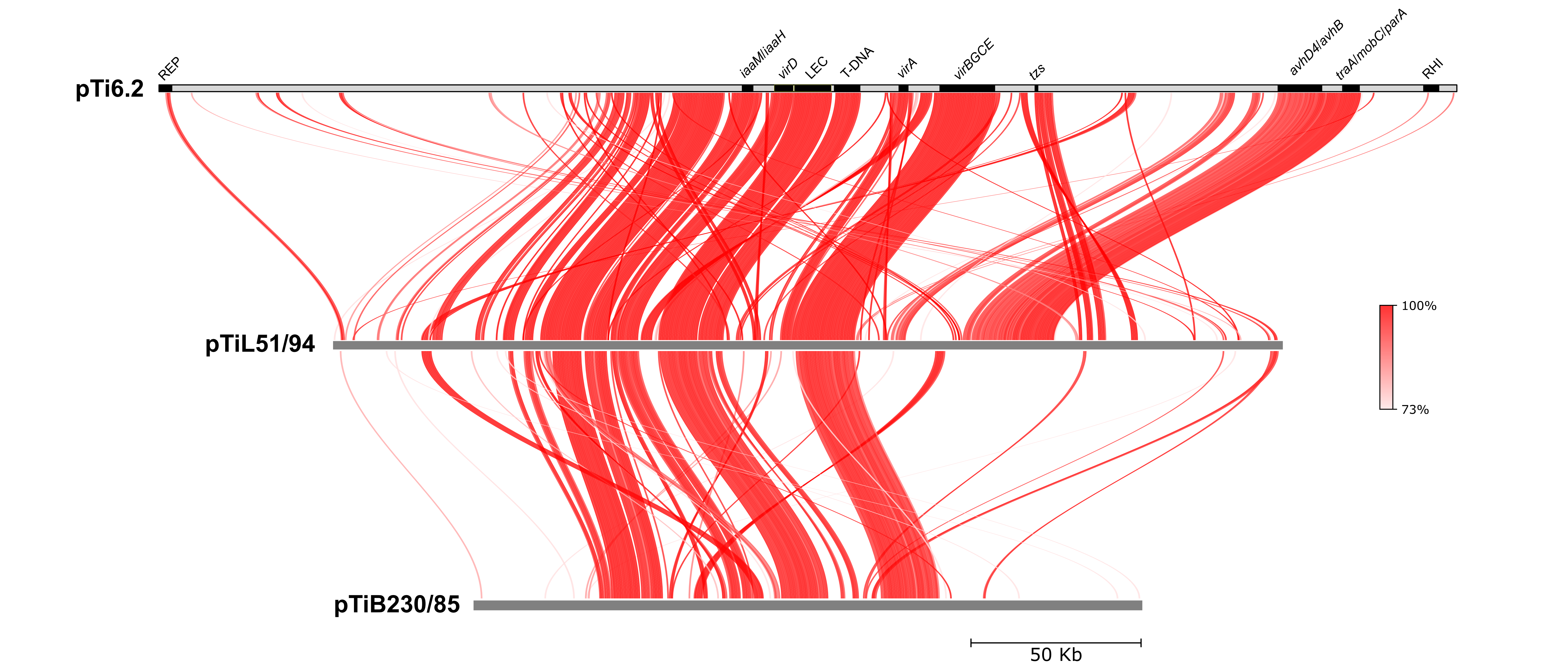

Fig. S2. Synteny between type VII (**a**) and type VIII (**b**) Ti plasmids. Orthologous mappings (500 bp fragment length) were computed with FastANI and plotted using the Python script visualize.py (https://github.com/moshi4/pyGenomeViz/tree/main/notebooks/fastANI). Each red line segment denotes an orthologous mapping between two replicons, indicating conserved regions. The darker color indicates a higher percentage of identity (see the legend on the right). Plasmid functional modules and specific genes are indicated as described in the Figure 1.

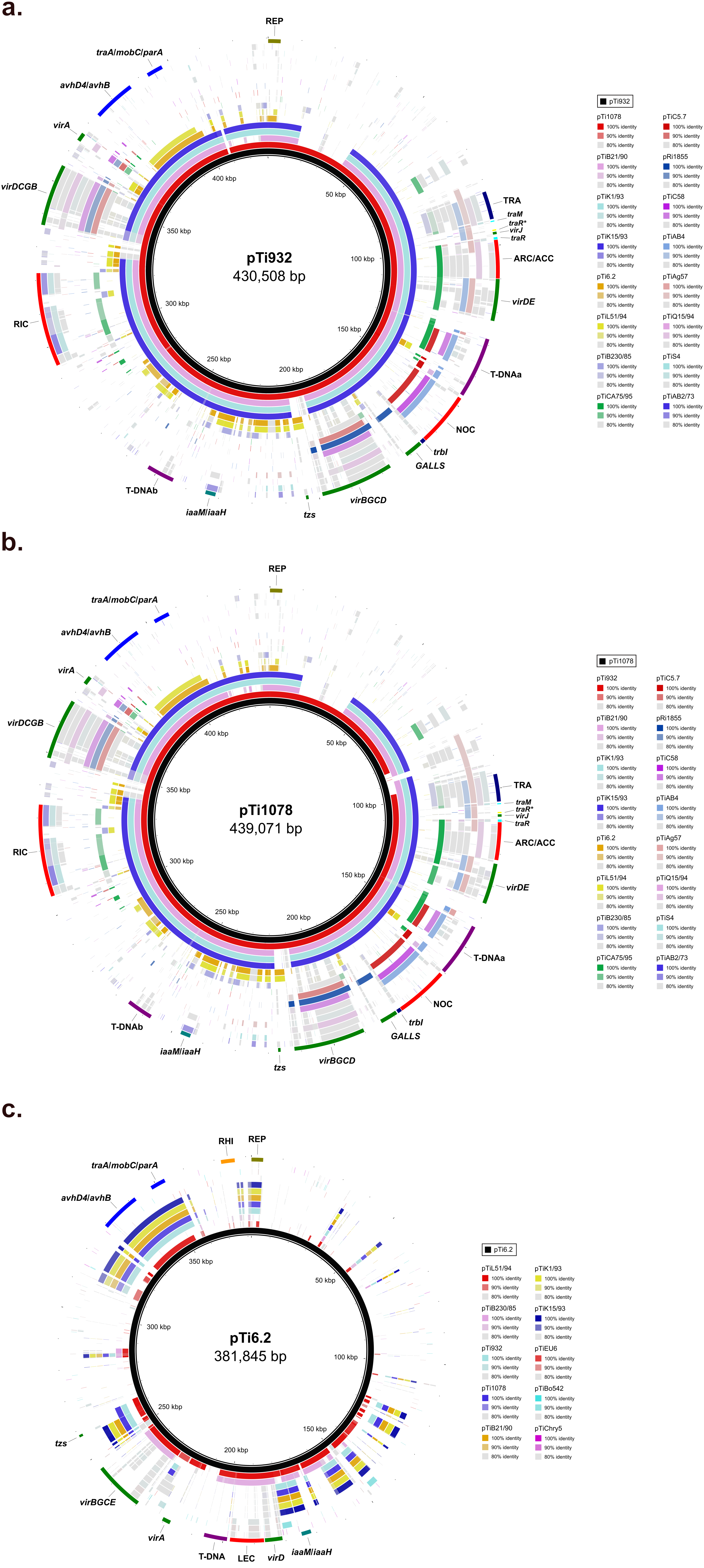

Fig. S3. Comparison of pTi932 (**a**), pTi1078 (**b**) and pTi6.2 (**c**) (reference sequences) with reference Ti/Ri plasmids (Table S1) (query sequences). Genetic coordinates of the reference sequences are shown within the thin inner ring. The thick black rings portray corresponding reference sequences (pTi932, pTi1078 and pTi6.2). The colored rings portray query sequences, as indicated in the Figure legend. The outermost ring indicates plasmid functional modules and specific genes as described in the Figure 1. The Figure was generated using BRIG software and edited with Inkscape.

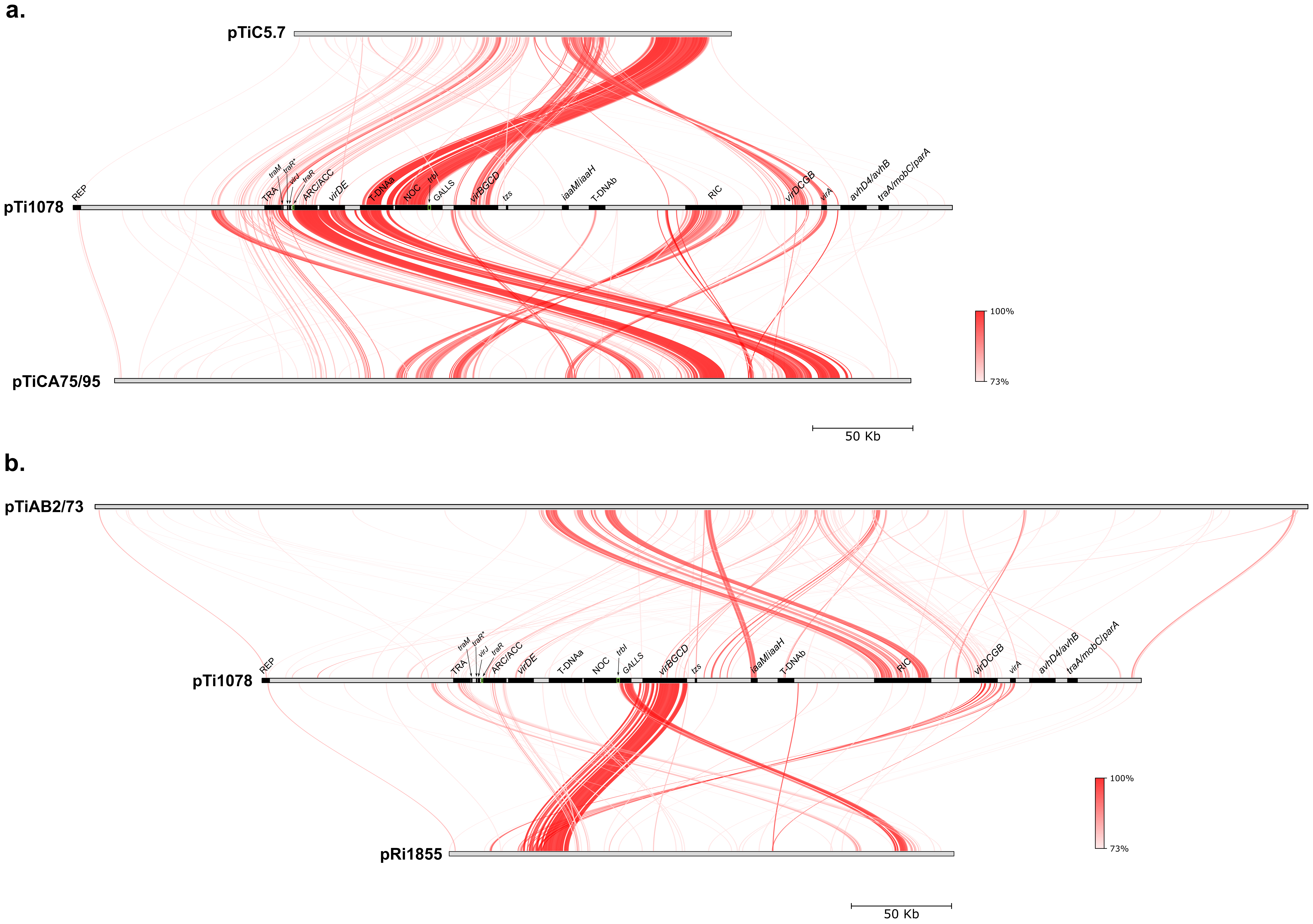

Fig. S4. Synteny analysis of pTi1078 and reference Ti/Ri plasmids. Plasmid pTi1078 was compared with plasmids pTiC5.7 and pTiCA75/95 (**a**), as well as with plasmids pTiAB2/73 and pRi1855 (**b**). Orthologous mappings (500 bp fragment length) were computed with FastANI and plotted using the Python script visualize.py (https://github.com/moshi4/pyGenomeViz/tree/main/notebooks/fastANI). Each red line segment denotes an orthologous mapping between two replicons, indicating conserved regions. The darker color indicates a higher percentage of identity (see the legend on the right). Plasmid functional modules and specific genes are indicated as described in the Figure 1.

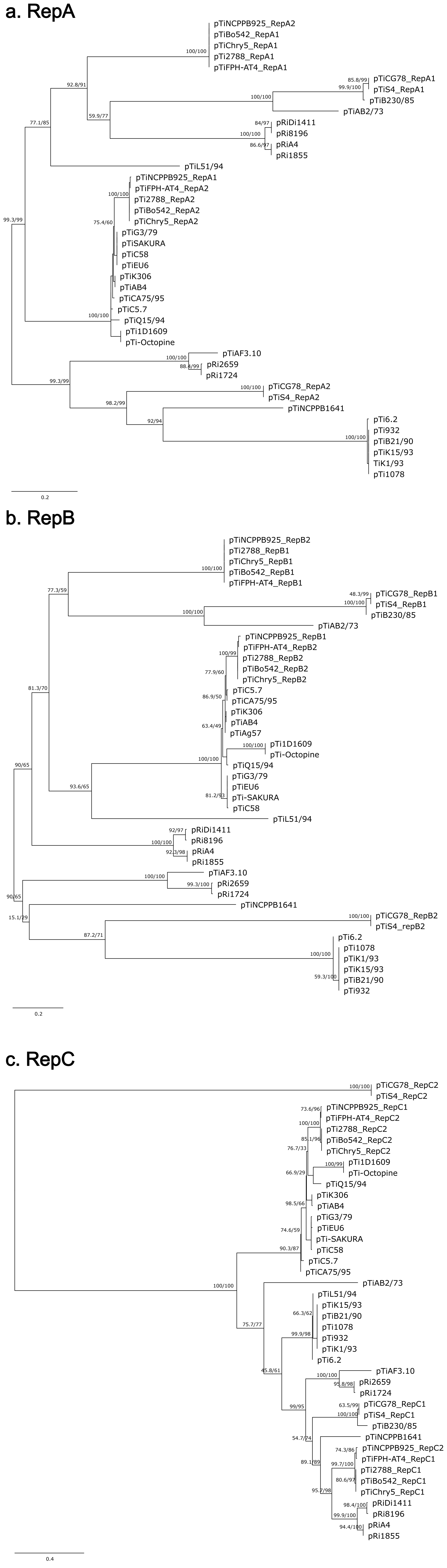

Fig. S5. Maximum likelihood tree based on RepA (**a**), RepB (**b**) and RepC (**c**) proteins of "tumorigenes" and reference Ti/Ri plasmids (Table S1). The tree was constructed using models LG+G4 (RepA and RepC) and LG+I+G4 (RepB). The numbers on the nodes indicate the SH-aLRT support values (first value) and ultra-fast bootstrap values (second value). The tree was midpoint rooted. The scale bar represents the estimated number of amino acid substitutions per site.

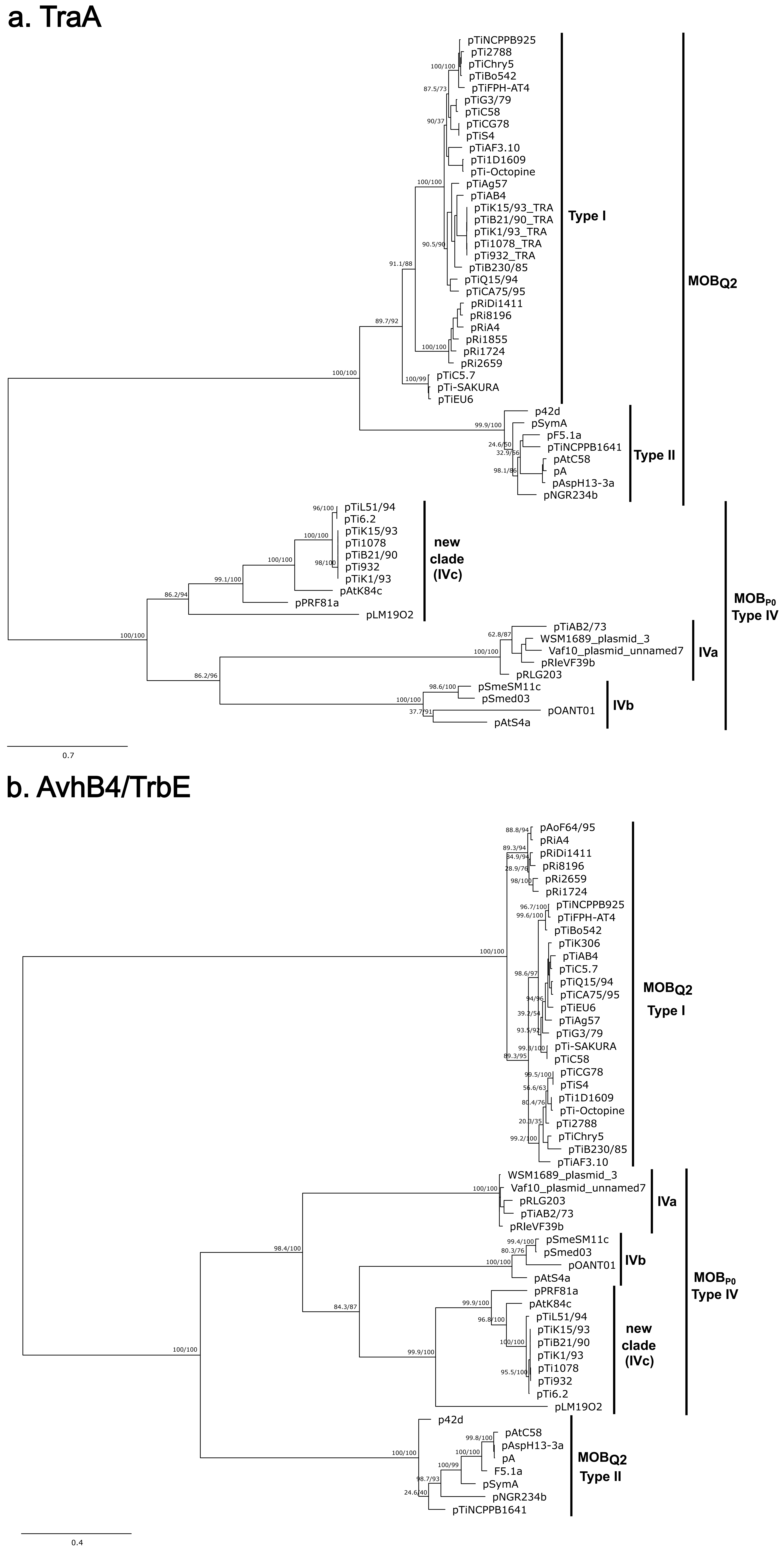

Fig. S6. Maximum likelihood tree based on TraA (**a**) and AvhB4/TrbE (**b**) proteins of "tumorigenes" and reference Ti/Ri plasmids (Table S1). The tree was constructed using models JTT+F+G4 (TraA) and LG+F+G4 (AvhB4/TrbE). The numbers on the nodes indicate the SH-aLRT support values (first value) and ultra-fast bootstrap values (second value). The tree was midpoint rooted. The scale bar represents the estimated number of amino acid substitutions per site.

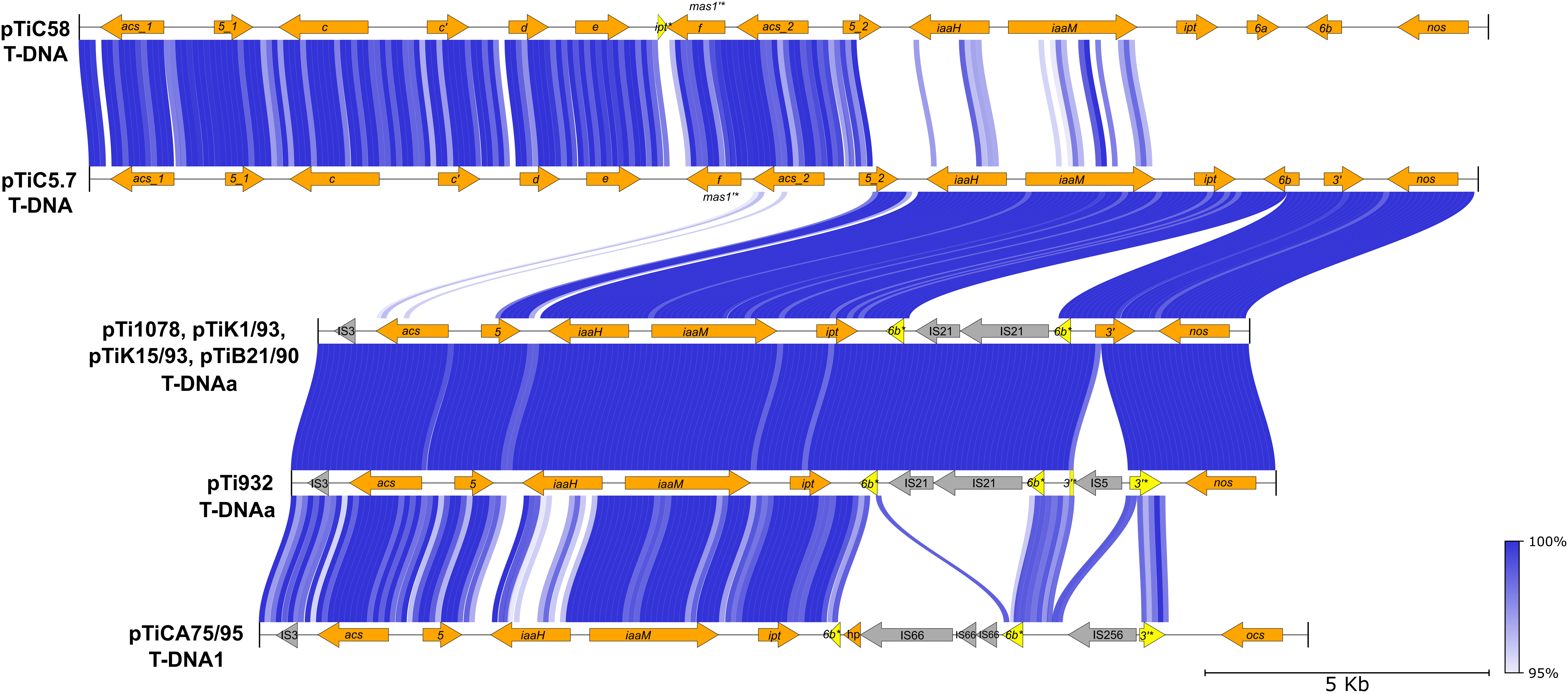

Fig. S7. Synteny and comparative analysis of T-DNAa of type VII Ti plasmids, including related T-DNA structures. Orthologous mappings (100 bp fragment length) were computed with FastANI and plotted using the Python script pgv-fastani.py (https://github.com/moshi4/pyGenomeViz/tree/main/notebooks/fastANI). Each red line segment denotes an orthologous mapping between two replicons, indicating conserved regions. The darker color indicates a higher percentage of identity (see the legend on the right). The colored arrows represent CDSs: intact genes (orange arrows), interrupted genes (yellow arrows), and insertion sequence (IS) elements (gray arrows). Yellow rectangles represent non-functional gene-fragments. Gene names are indicated inside the arrows. The name hp corresponds to genes encoding hypothetical proteins. Gene names with asterisks indicate interrupted genes.

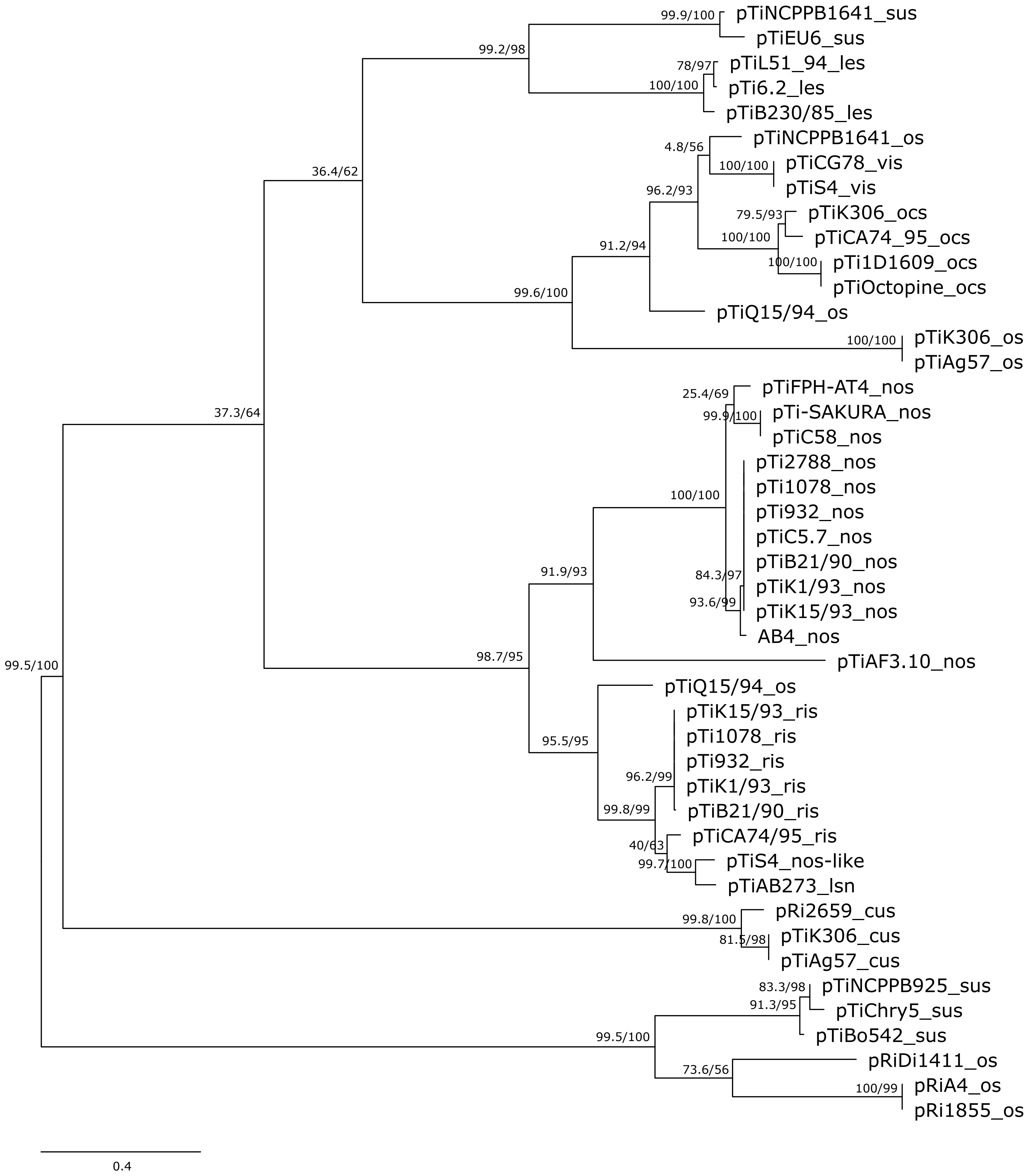

Fig. S8. Maximum likelihood tree based on opine synthase (*os*) proteins of "tumorigenes" and reference Ti/Ri plasmids (Table S1). The tree was constructed using model WAG+G4. The numbers on the nodes indicate the SH-aLRT support values (first value) and ultra-fast bootstrap values (second value). The tree was midpoint rooted. The scale bar represents the estimated number of amino acid substitutions per site.

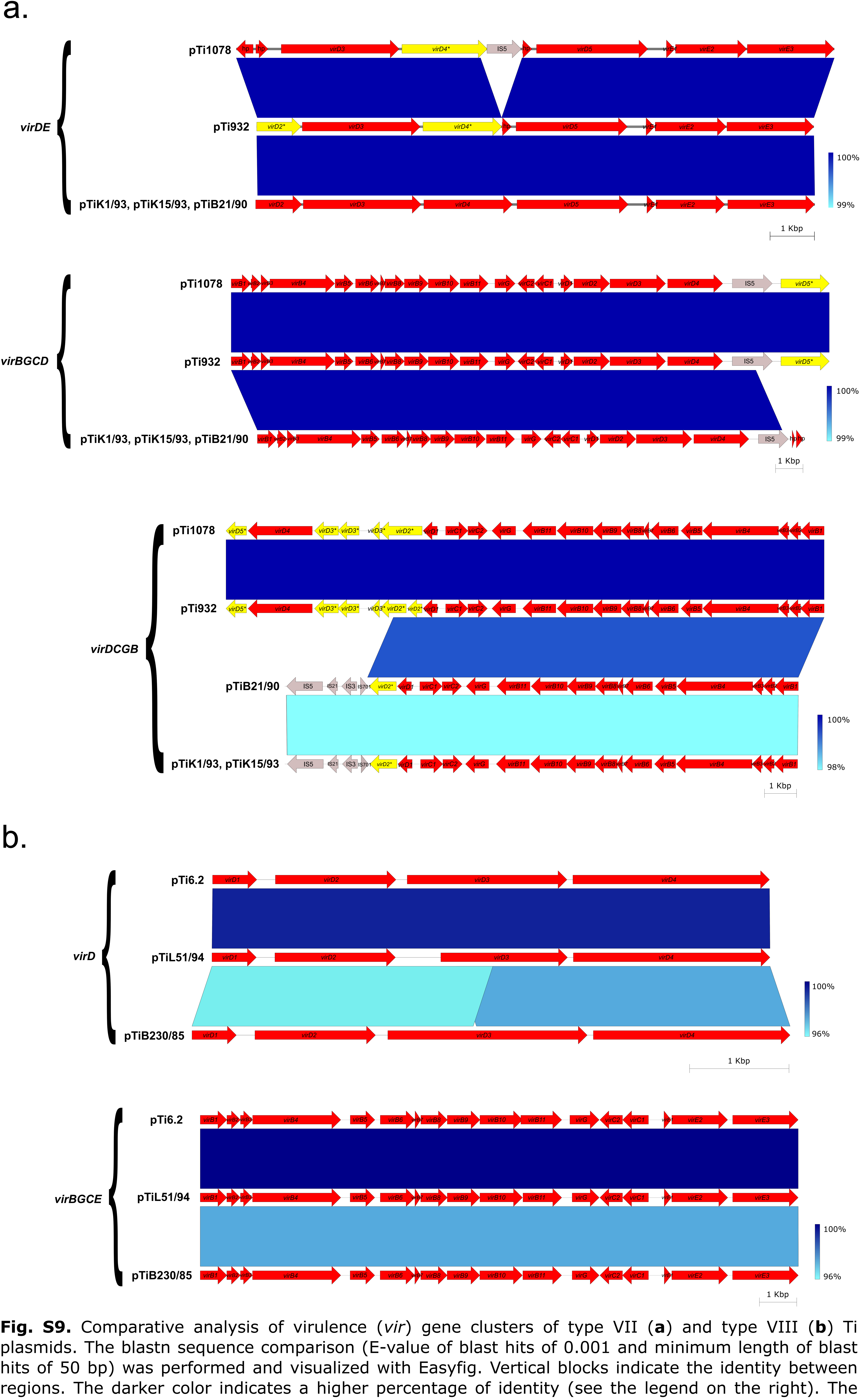

Fig. S9. Comparative analysis of virulence (*vir*) gene clusters of type VII (**a**) and type VIII (**b**) Ti plasmids. The blastn sequence comparison (E-value of blast hits of 0.001 and minimum length of blast hits of 50 bp) was performed and visualized with Easyfig. Vertical blocks indicate the identity between regions. The darker color indicates a higher percentage of identity (see the legend on the right). The colored arrows represent CDSs: intact genes (red arrows), interrupted genes (yellow arrows), and insertion sequence (IS) elements (gray arrows). Gene names are indicated inside the arrows. The name hp corresponds to genes encoding hypothetical proteins. Gene names with asterisks indicate interrupted genes.

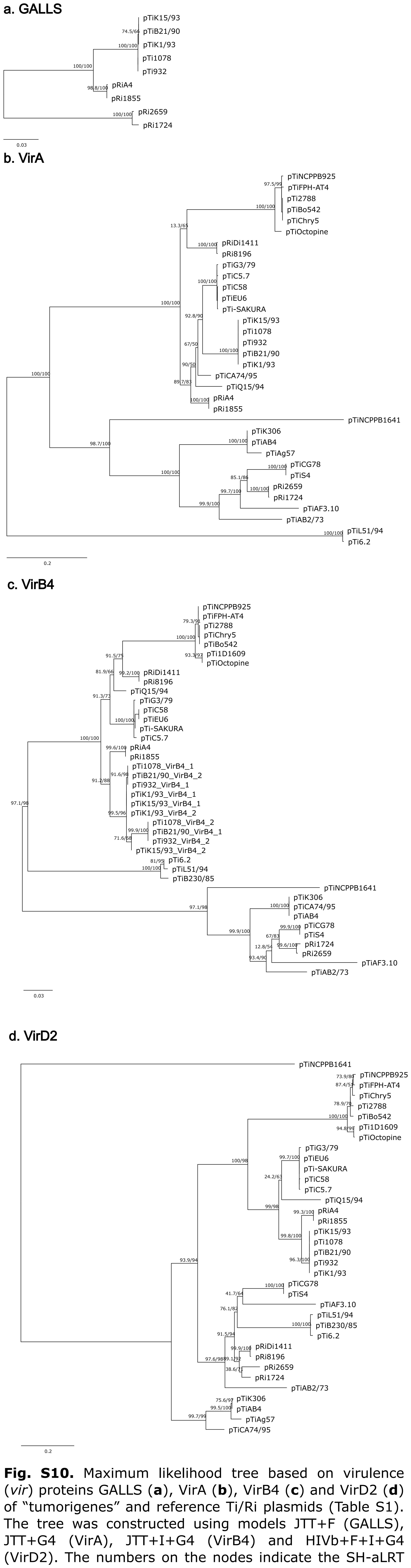

Fig. S10. Maximum likelihood tree based on virulence (*vir*) proteins GALLS (**a**), VirA (**b**), VirB4 (**c**) and VirD2 (**d**) of "tumorigenes" and reference Ti/Ri plasmids (Table S1). The tree was constructed using models JTT+F (GALLS), JTT+G4 (VirA), JTT+I+G4 (VirB4) and HIVb+F+I+G4 (VirD2). The numbers on the nodes indicate the SH-aLRT support values (first value) and ultra-fast bootstrap values (second value). The tree was midpoint rooted. The scale bar represents the estimated number of amino acid substitutions per site.

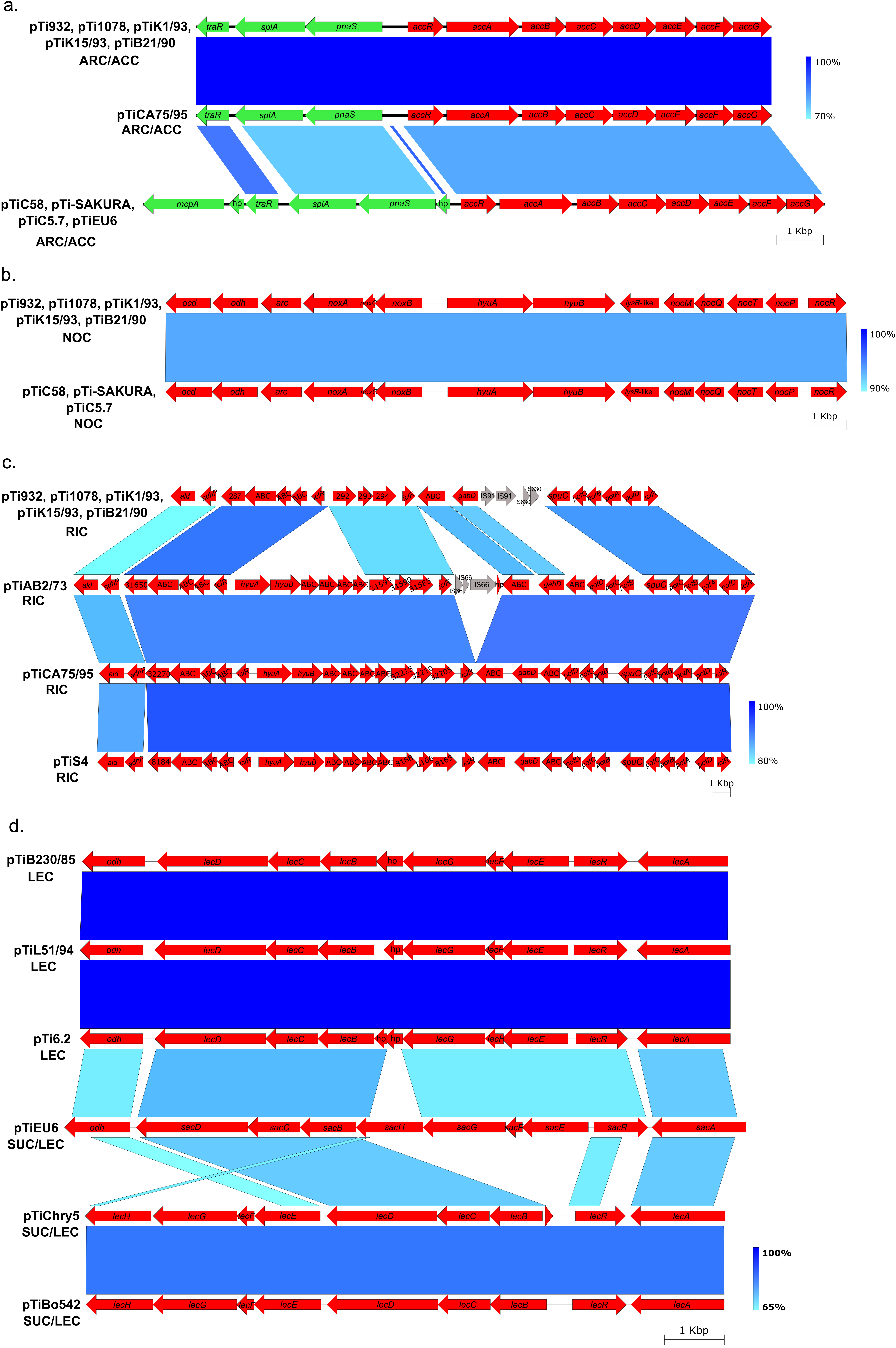

Fig. S11. Comparative analysis of regions associated with transport and catabolism of opines: agrocinopine (ACC) (**a**), which is linked to the region associated with agrocinopine regulation of conjugation (ARC); nopaline (NOC) (**b**); ridéopine (RIC) (**c**); and leucinopine (LEC) (**d**). The blastn sequence comparison (E-value of blast hits of 0.001 and minimum length of blast hits of 50 bp) was performed and visualized with Easyfig. Vertical blocks indicate the identity between regions. The darker color indicates a higher percentage of identity (see the legend on the right). The colored arrows represent CDSs: genes of the opine catabolic region (red arrows), genes of the region associated with agrocinopine regulation of conjugation (green arrows), and insertion sequence (IS) elements (gray arrows). Gene names are indicated inside the arrows. The name hp corresponds to genes encoding hypothetical proteins.

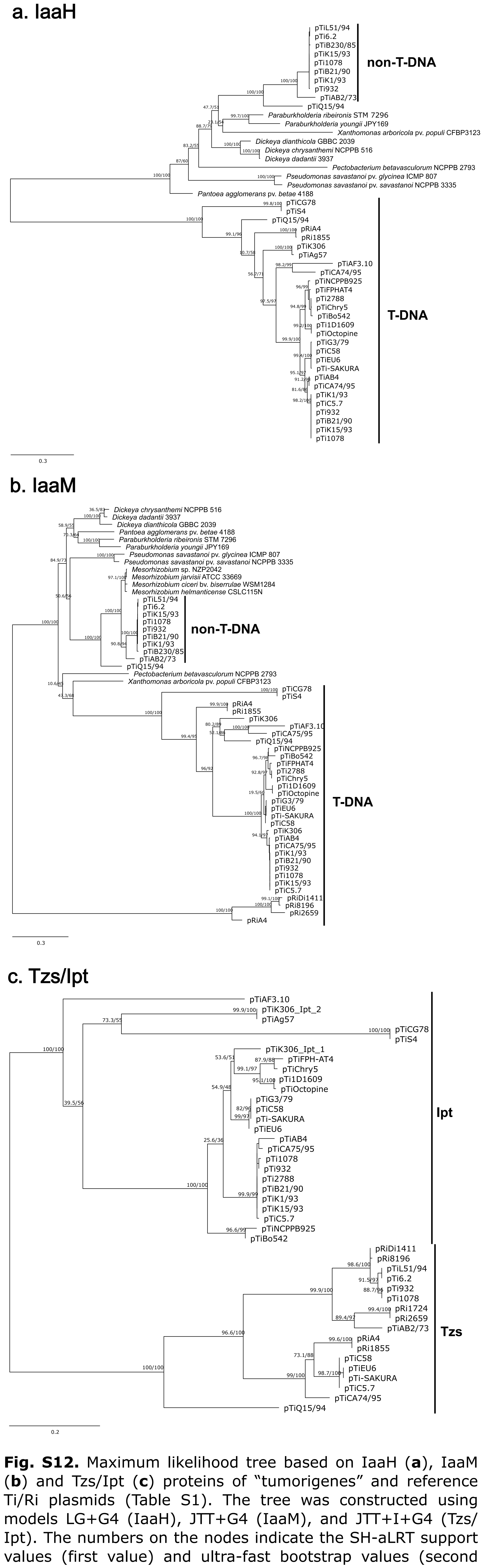

Fig. S12. Maximum likelihood tree based on IaaH (**a**), IaaM (**b**) and Tzs/Ipt (**c**) proteins of "tumorigenes" and reference Ti/Ri plasmids (Table S1). The tree was constructed using models LG+G4 (IaaH), JTT+G4 (IaaM), and JTT+I+G4 (Tzs/Ipt). The numbers on the nodes indicate the SH-aLRT support values (first value) and ultra-fast bootstrap values (second value). The tree was midpoint rooted. The scale bar represents the estimated number of amino acid substitutions per site.

a.

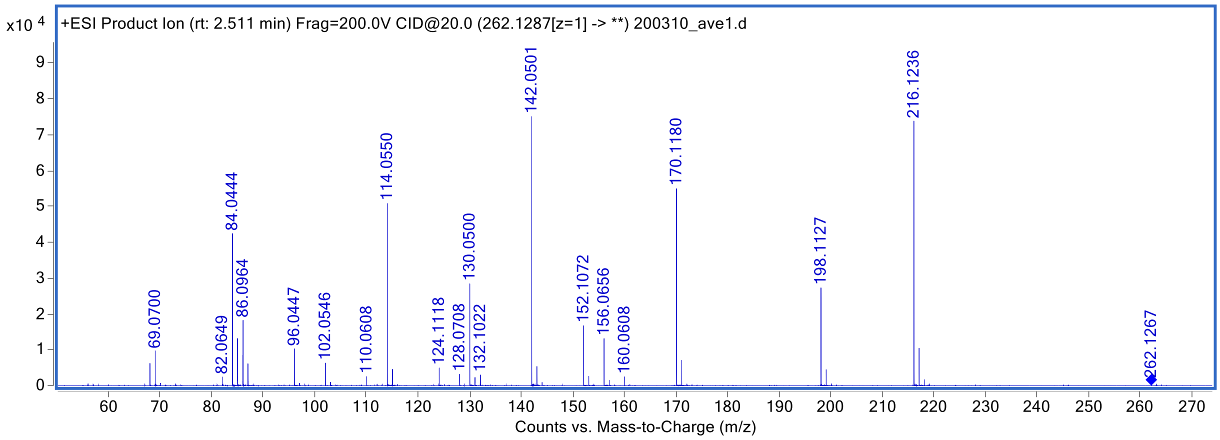

b.

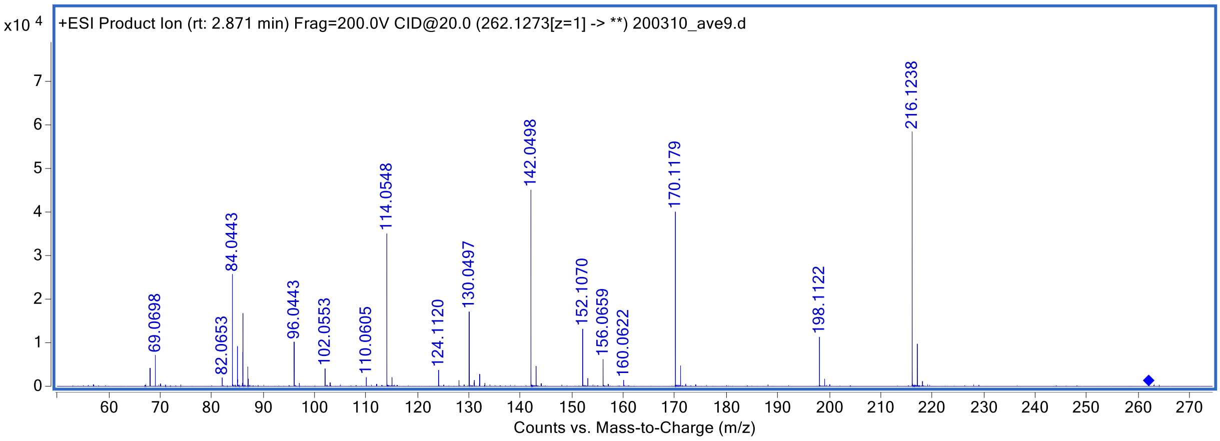

Fig. S13. MS/MS spectra of L,L-leucinopine (a) and putative D,L-leucinopine (b) detected in tomato tumor samples induced by *A. tumefaciens* Chry5 or *R. rhododendri* rho-6.2, respectively.
